# Palladium-Catalyzed Cross-Coupling of *Gem*-Bromofluoroalkenes with Alkylboronic Acids for the Synthesis of Alkylated Monofluoroalkenes

**DOI:** 10.3390/molecules25235532

**Published:** 2020-11-25

**Authors:** Laëtitia Chausset-Boissarie, Nicolas Cheval, Christian Rolando

**Affiliations:** Univ. Lille, CNRS, USR 3290—MSAP—Miniaturisation Pour la Synthèse, l’Analyse et la Protéomique, F-59000 Lille, France; nicolas.cheval@gmail.fr

**Keywords:** monofluoroalkenes, *gem*-bromofluoroalkenes, alkylboronic acids, Suzuki–Miyaura–cross-coupling

## Abstract

Monofluoroalkenes are versatile fluorinated synthons in organic synthesis, medicinal chemistry and materials science. In light of the importance of alkyl-substituted monofluoroalkenes efficient synthesis of these moieties still represents a synthetic challenge. Herein, we described a mild and efficient methodology to obtain monofluoroalkenes through a stereospecific palladium-catalyzed alkylation of *gem*-bromofluoroalkenes with primary and strained secondary alkylboronic acids under mild conditions. This novel strategy gives access to a wide range of functionalized tri- and tetrasubstituted monofluoroalkenes in high yield, with good functional group tolerance, independently from the *gem*-bromofluoroalkenes geometry.

## 1. Introduction

The incorporation of fluorine atoms into bioactive molecules hugely impacts their physicochemical and pharmacokinetic properties, prevents oxidative metabolism and, more important, modulates their overall biological activities [1,2]. Accordingly, fluorinated compounds are abundant scaffolds found in a large variety of materials, agrochemicals and pharmaceuticals [3,4,5,6,7,8]. In particular, monofluoroalkenes are highly valuable fluorinated synthons in organic synthesis, in high-performance materials and in medicinal chemistry as they are excellent peptide bond mimics with enhanced stability towards proteases and stable conformation, improving the molecule stability and lipophilicity [9,10]. Despite the importance of alkylated monofluoroalkenes, limited methodologies have been developed for their modular synthesis. Pioneering studies to obtain alkyl-substituted monofluoroalkenes were focused on classical olefination (Wittig, Horner-Wadsworth-Emmons or Julia Kocienski reaction) [11,12], electrophilic fluorination or fluorination of alkynes [13,14,15,16]. More recently, transition metal-catalyzed defluorinative alkylation of *gem*-difluoroalkenes [17,18,19,20,21] or *gem*-difluorocyclopropanes [22,23,24] with various carbon nucleophiles has proven to be efficient strategies to access alkylated monofluoroalkenes. In the meantime, photoredox monofluoroalkenylation of *gem*-difluoroalkenes has also been successfully applied for their syntheses [25,26,27,28]. Despite these remarkable achievements, defluorinative cross-coupling towards the C(*sp^3^*)-C(*sp^2^*) bond formation is still limited by the use of expensive catalytic systems, moderate *Z*/*E* selectivity, air-sensitive reagents or specific alkyl sources bearing a heteroatom at the α-position. *Gem*-bromofluoroalkenes, which are easily accessible, starting materials from aldehyde or ketones via a Wittig-Burton reaction, can also be efficient substrates for the selective formation of alkyl-substituted monofluoroalkenes [29,30]. In this regard, Pannecoucke’s group reported the selective synthesis of stereo-defined butylated *Z*-(fluoro)alkene by Pd-catalyzed cross-coupling of (*E*/*Z*)-*gem*-bromofluoroalkenes with an in situ-generated organozinc intermediate [31]. Following up, the group of Wnuk reported an elegant pallado-catalyzed Negishi cross-coupling of *gem*-bromofluoroalkenes with alkyl organozinc derivatives as coupling partners to selectively produce (*Z*)-monofluoroalkenes [32]. Nevertheless, one of the drawbacks of these pathways is a low functional group tolerance and the use of sensitive reagents. Therefore, despite great successes achieved, the development of mild and practical methodologies to monofluoroalkenes, especially 2-fluoroalkyl scaffolds, remains an appealing task. Continuing our research directed towards the development of new methodology for the synthesis of functionalized monofluoroalkenes [33,34,35,36] Herein, we report the first example of a stereospecific Suzuki-Miyaura-cross-coupling reaction with readily available alkyl boronic acids that is adaptable across a range of *gem*-bromofluoroalkenes providing a large array of alkylated monofluoroalkenes with retention of configuration and in good yields under mild conditions.

## 2. Results and Discussion

At the outset of the study, coupling reactions were investigated with the easily accessible (*E*/*Z*)-1-(2-bromo-2-fluorovinyl)-4-nitrobenzene **1a** [31] and butylboronic acid **2a**.

To establish the best reaction conditions, a broad range of palladium catalyst precursors, bases, solvents, temperatures and phosphine ligands were evaluated (Table 1). An initial survey demonstrated that the use of PdCl_2_dppf as catalyst gave the desired product as a mixture of *E*/*Z* isomers in 95% yield (entries 1–3). Among the bases, Cs_2_CO_3_ proved to be the most effective (entries 3–7). Subsequently, the solvents were screened, and the original biphasic mixture of toluene/H_2_O (9:1) was the best of choice (entries 3, 8–10). Further examination revealed that a decrease in the reaction temperature reduces the reaction efficiency (entries 11–12). Common ligands of palladium were tested (entries 13–17), and bidentate bisphosphines and, above all, those with large P-Pd-P bite angles appeared to be essentials [37]. Under some conditions, (*E*)-isomer reacts faster than the corresponding (*Z*)-isomers in Pd-catalyzed coupling reactions (entries 3, 5, 16). The best catalytic system was found to be Pd_2_(dba)_3_ (2 mol%) with xantphos (2 mol%) as the catalyst and Cs_2_CO_3_ as the base in a mixture of toluene/H_2_O (9:1) at 80 °C under nitrogen affording the desired product in almost quantitative yield (entry 15).

**Scheme 1:**
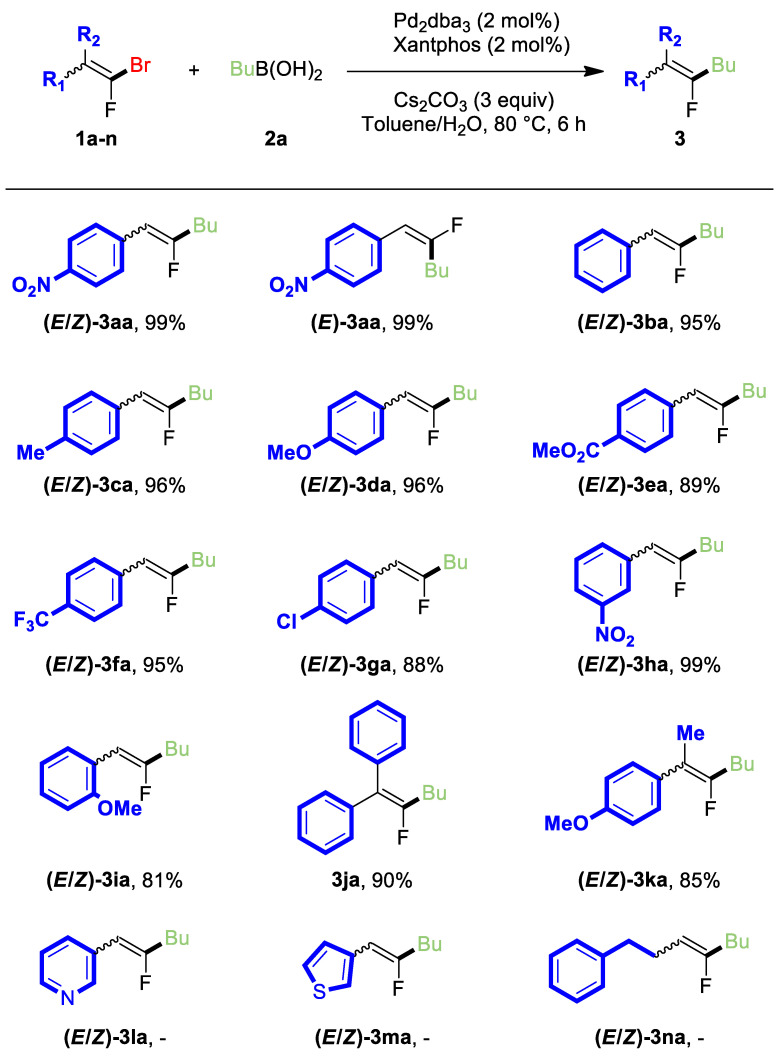
Palladium-catalyzed cross-coupling of butylboronic acid **2a** with various *gem*-bromofluoroalkenes **1a**–**n**.

With the optimized conditions in hand, we investigated the substrate scope of the cross-coupling reaction on the *gem*-bromofluoroalkene part (Scheme 1). A large range of (*E*/*Z*)-*gem*-bromofluoroalkenes was successfully cross-coupled to afford the related *Z* and *E* monofluoroalkenes in good to excellent isolated yield. The electronic effects of the substituents on the aromatics rings showed no obvious influence on this transformation since (*E*/*Z*)-*gem*-bromofluoroalkenes possessing electron neutral (**3ba**–**ca**), electron-donating (**3da**) and electron-withdrawing groups (**3ea**–**ga**) provided the (*E*/*Z*)-monofluoroalkenes in high yields. Several sensitive or valuable functional groups, notably for further post-functionalizations, such as esters, trifluoromethyl and nitro groups, were well tolerated throughout the coupling reactions. In all cases, no sterodifferentiation was observed since a mixture of the corresponding *E*/*Z* isomers was obtained with the same isomeric composition of the starting material. The cross-coupling reaction of isomerically pure (*Z*)-1-bromo-1-fluoroalkene **1a** led stereospecifically to a corresponding (*E*)-monofluoroalkene **3aa** with complete retention of the stereochemistry confirming the stereospecificity of the reaction. Interestingly, *gem*-bromofluoroalkene that are meta-substituted (**3ha**) or sterically hindered at the ortho position (**3ia**) were suitable coupling partners for the reaction albeit, aryl *gem*-bromofluoroalkenes bearing substituents in the para position showed better reactivity. Gratifyingly, in the case of symmetric and unsymmetric *gem*-bromofluoroalkenes derived from ketones, the corresponding tetrasubstituted monofluoroalkenes **3ja** and **3ka** are obtained in excellent isolated yield. Unfortunately, the reaction is not compatible with nitrogen or sulfur hetaryl *gem*-bromofluoroalkenes (**3la**–**ma**) mainly due to the degradation of the starting material. In addition, when alkylated *gem*-bromofluoroolefin **1n** was used as the substrate, the reaction also failed to give any coupling product.

**Scheme 2:**
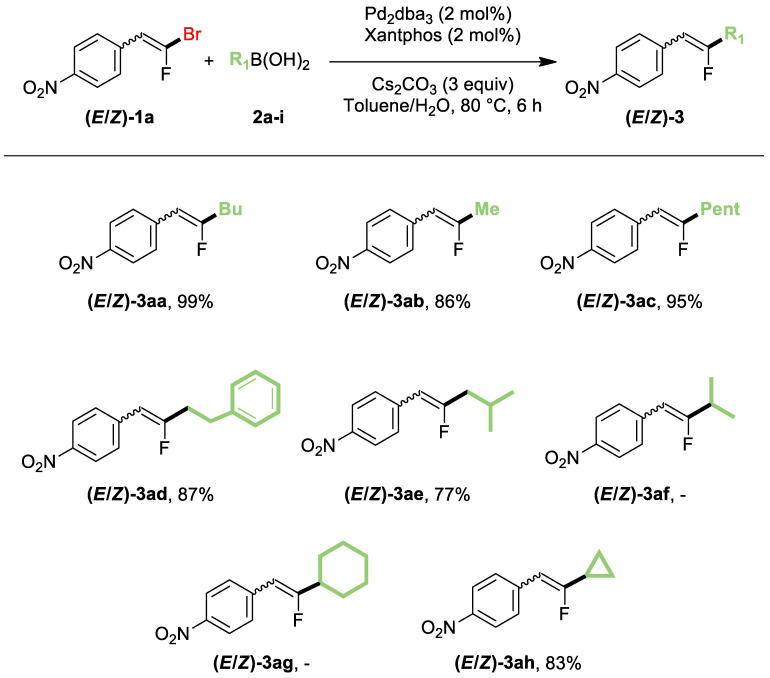
Palladium-catalyzed cross-coupling of (*E*/*Z*)-1-(2-bromo-2-fluorovinyl)-4-nitrobenzene **1a** with various alkyl boronic acids **2a**–**i**.

We then examined the coupling reactions with different primary and secondary alkyl boronic acids and (*E*/*Z*)-1-(2-bromo-2-fluorovinyl)-4-nitrobenzene 1a using the same set of reaction conditions developed (Scheme 2). All of the primary aliphatic alkyl boronic acids 2a–e provided the desired product 3aa-ae in good to excellent isolated yields. In the case of secondary alkyl substituents such as isopropyl or cyclohexyl boronic acids 2f–g, no reaction occurred; only starting materials were recovered. Cyclopropyl boronic acid 2 h was shown to undergo a cross-coupling reaction giving the product in 83% yield. This could be due to the geometry of the substrate, which suppresses β-hydride elimination.

## 3. Materials and Methods

### 3.1. General Methods

All reagents were purchased from commercial suppliers and were used without further purification unless otherwise indicated. Thin-layer chromatography (TLC) was performed on silica gel 60 F254 plates (Merck, Pfizer, Sanofi) and visualized under UV (254 nm) or by staining with potassium permanganate or phosphomolybdic acid. The purification of the obtained products was performed by flash chromatography using silica gel (230–400 mesh, 0.040–0.063 mm).

NMR spectra were recorded on a Bruker AVANCE 300 spectrometer (Bruker Corporation, Billerica, MA, USA) at 300 MHz (75 MHz). Chemical shifts are given in parts per million relative to the solvent signal. Multiplicities of the signals are reported using the standard abbreviations: singlet (s), doublet (d), triplet (t), quartet (q) and multiplet (m). Coupling constants are reported in hertz (Hz). Coupling constants (J) are reported in hertz (Hz). High-resolution mass spectra (HRMS) were performed on a ThermoFisher Scientific LTQ Orbitrap XL mass spectrometer (Thermo Fischer Scientific, Bremen, Germany) using electrospray ionization (ESI).

### 3.2. Synthesis of Gem-Bromofluoroalkenes ***1***

Gem-bromofluoroalkenes **1a**–**n** were synthesized according to known procedures reported by Pannecoucke’s group^31^ from the appropriate aldehyde and tribromofluoromethane.

### 3.3. General Procedure for the Synthesis of Monofluoroalkenes ***3***

In a Schlenk tube, *gem*-bromofluoroalkene **1** (1.0 equiv), boronic acid **2** (1.2 equiv), Pd_2_dba_3_·CHCl_3_ (2 mol%), xantphos (2 mol%) and Cs_2_CO_3_ (3 equiv) were added. The vial was flushed under nitrogen, then filled with a mixture of toluene/H_2_O (9:1) (0.09 M). The reaction mixture was heated for 6 h at 80 °C then cooled to r.t., filtered through Celite^®^ and washed with EtOAc. The filtrate was concentrated under vacuum, and the residue was purified by flash chromatography on silica gel (petroleum ether/EtOAc = 100:0 to 95:5) to afford the pure product **3**. ^1^H-, ^13^C- and ^19^F-NMR spectra of products can be found in Supplementary Materials.

#### 3.3.1. (*E*/*Z*)-1-(2-fluorohex-1-en-1-yl)-4-nitrobenzene (**3aa**)

(*E*/*Z*)-1-(2-bromo-2-fluorovinyl)-4-nitrobenzene **1a** (0.23 mmol, 56.32 mg), butyl boronic acid **2a** (0.28 mmol, 28.45 mg), Pd_2_dba_3_·CHCl_3_ (4.710^−3^ mmol, 4.25 mg), xantphos (4.710^−3^ mmol, 2.69 mg), Cs_2_CO_3_ (0.70 mmol, 227.10 mg), toluene/H_2_O (2.53 mL) were reacted according to general procedure. The crude product was purified by silica gel column chromatography (petroleum ether/EtOAc = 100:0 to 95:5) affording compound **3aa** in 99% yield (50.80 mg) as a yellow solid. ^1^H NMR (300 MHz, CDCl_3_): δ 8.09 (t, *J* = 9.1 Hz, 2.0H), 7.51 (d, *J* = 8.9 Hz, 0.8 H), 7.26 (d, *J* = 8.4 Hz, 1.2H), 6.15 (d, *J^E^*_H-F_ = 21.1 Hz, 0.6H), 5.49 (d, *J^Z^*_H-F_ = 38.1 Hz, 0.4H), 2.47–2.23 (m, 2.0H), 1.59–1.50 (m, 2.0H), 1.37–1.27 (m, 2.0H), 0.90–0.81 (m, 3.0H). ^13^C NMR (75 MHz, CDCl_3_): δ 164.1 (d, *^1^J^E^_C-F_* = 257.5 Hz), 163.6 (d, *^1^J^Z^_C-F_* = 271.8 Hz), 145.3 (2), 140.5 (d, *^3^J^E^_C-F_* = 14.8 Hz), 139.6 (d, *^3^J^Z^_C-F_* = 2.7 Hz), 127.9 (2), 127.7, 127.6, 122.8 (2), 122.7 (2), 106.1 (d, *^2^J^Z^_C-F_* = 31.0 Hz), 103.5 (d, *^2^J^E^_C-F_* = 8.2 Hz), 31.9 (d, *^2^J^Z^_C-F_* = 25.6 Hz), 28.0 (d, *^2^J^E^_C-F_* = 26.6 Hz), 27.3 (2), 21.2, 21.0, 12.7 (2). ^19^ F NMR (282.5 MHz, CDCl_3_): δ − 99.23 (q, *J^E^* = 23.2 Hz), −101.65 (dt, *J^Z^* = 39.7, 18.1 Hz). HRMS (ESI): *m*/*z* [M + H]^+^ calc. for C_12_H_15_FNO_2_, 224.1081 found 224.1080.

#### 3.3.2. (*E*)-1-(2-fluorohex-1-en-1-yl)-4-nitrobenzene (***E*-3aa**)

(*Z*)-1-(2-bromo-2-fluorovinyl)-4-nitrobenzene **1a** (0.23 mmol, 56.32 mg), butyl boronic acid **2a** (0.28 mmol, 28.45 mg), Pd_2_dba_3_·CHCl_3_ (4.710^−3^ mmol, 4.25 mg), xantphos (4.710^−3^ mmol, 2.69 mg), Cs_2_CO_3_ (0.70 mmol, 227.10 mg), toluene/H_2_O (2.53 mL) were reacted according to general procedure. The crude product was purified by silica gel column chromatography (petroleum ether/EtOAc = 100:0 to 95:5) affording compound *E-***3aa** in 99% yield (50.80 mg) as a yellow solid. ^1^H NMR (300 MHz, CDCl_3_): δ 8.09 (t, *J* = 9.1 Hz, 2.0H), 7.51 (d, *J* = 8.9 Hz, 2.0 H), 6.15 (d, *J^E^*_H-F_ = 21.1 Hz, 1.0H), 2.47–2.23 (m, 2.0H), 1.59–1.50 (m, 2.0H), 1.37–1.27 (m, 2.0H), 0.90–0.81 (m, 3.0H). ^13^C NMR (75 MHz, CDCl_3_): δ 164.1 (d, *^1^J^E^_C-F_* = 257.5 Hz), 145.3, 140.5 (d, *^3^J^E^_C-F_* = 14.8 Hz), 127.9, 127.7, 122.8, 122.7, 103.5 (d, *^2^J^E^_C-F_* = 8.2 Hz), 28.0 (d, *^2^J^E^_C-F_* = 26.6 Hz), 27.3, 21.2, 12.7. ^19^ F NMR (282.5 MHz, CDCl_3_): δ − 99.23 (q, *J^E^* = 23.2 Hz). HRMS (ESI): *m*/*z* [M + H]^+^ calc. for C_12_H_15_FNO_2_, 224.1081 found 224.1082.

#### 3.3.3. (*E*/*Z*)-(2-fluorohex-1-en-1-yl)benzene (**3ba**)

(*E*/*Z*)-1-(2-bromo-2-fluorovinyl)-benzene **2b** (0.23 mmol, 46.39 mg), butyl boronic acid **2a** (0.28 mmol, 28.45 mg), Pd_2_dba_3_·CHCl_3_ (4.710^−3^ mmol, 4.25 mg), xantphos (4.710^−3^ mmol, 2.69 mg), Cs_2_CO_3_ (0.70 mmol, 227.10 mg), toluene/H_2_O (2.53 mL) were reacted according to general procedure. The crude product was purified by silica gel column chromatography (petroleum ether/EtOAc = 100:0 to 95:5) affording compound **3ba** in 95% yield (39.25 mg) as a yellow solid. ^1^H NMR (300 MHz, CDCl_3_): δ 7.43–7.34 (m, 1.00H), 7.28–7.20 (m, 2.00H), 7.16–7.05 (m, 2.00H), 6.10 (d, *J^E^*_H-F_ = 22.0 Hz, 0.53H), 5.38 (d, *J^Z^*_H-F_ = 39.5 Hz, 0.47H), 2.43–2.19 (m, 2.00H), 1.57–1.46 (m, 2.00H), 1.31 (ddd, *J* = 15.1, 9.5, 7.4 Hz, 2.00H), 0.87 (t, *J* = 6.3 Hz, 1.40H), 0.82 (t, *J* = 6.4 Hz, 1.60H). ^13^C NMR (75 MHz, CDCl_3_): δ 162.8 (d, *^1^J^E^_C-F_* = 251.3 Hz), 161.3 (d, *^1^J^Z^_C-F_* = 265.1 Hz), 134.5 (d, *^3^J^E^_C-F_* = 14.1 Hz), 134.0 (d, *^3^J^Z^_C-F_* = 2.5 Hz), 128.6, 128.6 (3), 128.5 (2), 128.4, 128.3, 126.7 (2), 108.1 (d, *^2^J^Z^_C-F_* = 28.6 Hz), 105.7 (d, *^2^J^E^_C-F_* = 8.8 Hz), 32.8 (d, *^2^J^Z^_C-F_* = 26.3 Hz), 28.7 (d, *^2^J^E^_C-F_* = 29.2 Hz), 28.5 (2), 22.3, 22.1, 13.8 (2).^19^ F NMR (282 MHz, CDCl_3_): δ − 98.35 (dd, *J^E^* = 45.7, 23.4 Hz), −100.61 (dt, *J^Z^* = 39.4, 18.0 Hz). HRMS (ESI): *m*/*z* [M + H]^+^ calc. for C_12_H_16_F 179.1230, found 179.1233.

#### 3.3.4. (*E*/*Z*)-1-(2-fluorohex-1-en-1-yl)-4-methylbenzene (**3ca**)

(*E*/*Z*)-1-(2-bromo-2-fluorovinyl)-4-methylbenzene **2c** (0.23 mmol, 49.64 mg), butyl boronic acid **2a** (0.28 mmol, 28.45 mg), Pd_2_dba_3_·CHCl_3_ (4.710^−3^ mmol, 4.25 mg), xantphos (4.710^−3^ mmol, 2.69 mg), Cs_2_CO_3_ (0.70 mmol, 227.10 mg), toluene/H_2_O (2.53 mL) were reacted according to general procedure. The crude product was purified by silica gel column chromatography (petroleum ether/EtOAc = 100:0 to 95:5) affording compound **3ca** in 96% yield (42.79 mg) as a yellow solid. ^1^H NMR (300 MHz, CDCl_3_): δ 7.39 (d, *J* = 8.2 Hz, 1.00H), 7.21–7.08 (m, 3.00H), 6.18 (d, *J^E^*_H-F_ = 22.1 Hz, 0.51H), 5.45 (d, *J^Z^*_H-F_ = 39.8 Hz, 0.49H), 2.54–2.29 (m, 2.00H), 2.36 (s, 1.47H), 2.35 (s, 1.53H), 1.64–1.56 (m, 2.00H), 1.47–1.37 (m, 2.00H), 0.98 (t, *J* = 5.5 Hz, 1.47H), 0.93 (t, *J* = 5.5 Hz, 1.53H). ^13^C NMR (75 MHz, CDCl_3_): δ 162.5 (d, *^1^J^E^_C-F_* = 250.2 Hz), 160.9 (d, *^1^J^Z^_C-F_* = 263.9 Hz), 136.4 (2), 131.6 (d, *^3^J^E^_C-F_* = 14.0 Hz), 131.3 (d, *^3^J^Z^_C-F_* = 2.5 Hz), 129.3 (3), 129.2, 128.5, 128.4, 128.3, 128.2, 108.0 (d, *^2^J^Z^_C-F_* = 28.6 Hz), 105.6 (d, *^2^J^E^_C-F_* = 9.0 Hz), 32.9 (d, *^2^J^Z^_C-F_* = 26.4 Hz), 28.9 (d, *^2^J^E^_C-F_* = 28.6 Hz), 28.7 (2), 22.5, 22.2, 21.3, 21.2, 13.9 (2).^19^ F NMR (282 MHz, CDCl_3_): δ − 99.23 (q, *J^E^* = 23.2 Hz), −101.65 (dt, *J^Z^* = 39.7, 18.1 Hz). HRMS (ESI): *m*/*z* [M + H]^+^ calc. for C_13_H_18_F 193.1387, found 193.1388.

#### 3.3.5. (*E*/*Z*)-1-(2-fluorohex-1-en-1-yl)-4-methoxybenzene (**3da**)

(*E*/*Z*)-1-(2-bromo-2-fluorovinyl)-4-methoxybenzene **2d** (0.23 mmol, 53.35 mg), butyl boronic acid **2a** (0.28 mmol, 28.45 mg), Pd_2_dba_3_·CHCl_3_ (4.710^−3^ mmol, 4.25 mg), xantphos (4.710^−3^ mmol, 2.69 mg), Cs_2_CO_3_ (0.70 mmol, 227.10 mg), toluene/H_2_O (2.53 mL) were reacted according to general procedure. The crude product was purified by silica gel column chromatography (petroleum ether/EtOAc = 100:0 to 95:5) affording compound **3da** in 96% yield (46.35 mg) as a yellow solid. ^1^H NMR (300 MHz, CDCl_3_): δ 7.42 (d, *J* = 8.9 Hz, 0.96H), 7.13 (d, *J* = 8.3 Hz, 1.04H), 6.90–6.84 (m, 2.00H), 6.14 (d, *J^E^*_H-F_ = 22.0 Hz, 0.52H), 5.41 (d, *J^Z^*_H-F_ = 39.8 Hz, 0.48H), 3.81 (s, 3.00H), 2.49–2.28 (m, 2.00H), 1.64–1.56 (m, 2.00H), 1.45–1.36 (m, 2.00H), 0.96 (t, *J* = 5.4 Hz, 1.44H), 0.92 (t, *J* = 5.5 Hz, 1.56H). ^13^C NMR (75 MHz, CDCl_3_): δ 162.1 (d, *^1^J^E^_C-F_* = 249.3 Hz), 160.1 (d, *^1^J^Z^_C-F_* = 262.2 Hz), 158.6, 158.4, 129.7, 129.6 (2), 129.5, 126.9 (d, *^3^J^E^_C-F_* = 13.9 Hz), 126.8 (d, *^3^J^Z^_C-F_* = 2.3 Hz), 114.0 (4), 107.6 (d, *^2^J^Z^_C-F_* = 28.9 Hz), 105.1 (d, *^2^J^E^_C-F_* = 9.2 Hz), 55.4 (2), 32.9 (d, *^2^J^Z^_C-F_* = 26.5 Hz), 28.8 (d, *^2^J^E^_C-F_* = 27.1 Hz), 28.7 (2), 22.4, 22.2, 13.9 (2). ^19^ F NMR (282 MHz, CDCl_3_): δ − 100.27 (dd, *J^E^* = 45.9, 23.2 Hz), −103.58 (dt, *J^Z^* = 39.8, 18.3 Hz). HRMS (ESI): *m*/*z* [M + H]^+^ calc. for C_13_H_18_FO, 209.1336 found 209.1336.

#### 3.3.6. (*E*/*Z*)-methyl 4-(2-fluorohex-1-en-1-yl)benzoate (**3ea**)

(*E*/*Z*)-methyl 4-(2-bromo-2-fluorovinyl)benzoate **2e** (0.23 mmol, 59.84 mg), butyl boronic acid **2a** (0.28 mmol, 28.45 mg), Pd_2_dba_3_·CHCl_3_ (4.710^−3^ mmol, 4.25 mg), xantphos (4.710^−3^ mmol, 2.69 mg), Cs_2_CO_3_ (0.70 mmol, 227.10 mg), toluene/H_2_O (2.53 mL) were reacted according to general procedure. The crude product was purified by silica gel column chromatography (petroleum ether/EtOAc = 100:0 to 95:5) affording compound **3ea** in 89% yield (48.75 mg) as a yellow solid.^1^H NMR (300 MHz, CDCl_3_): δ 7.90 (dd, *J* = 8.4, 6.0 Hz, 2.00H), 7.43 (d, *J* = 8.5 Hz, 0.92H), 7.17 (d, *J* = 8.1 Hz, 1.08H), 6.12 (d, *J^E^*_H-F_ = 21.6 Hz, 0.54H), 5.43 (d, *J* = 38.9 Hz, 0.46H), 3.83 (s, 1.62H), 3.82 (s, 1.38H), 2.44–2.21 (m, 2.00H), 1.57–1.48 (m, 2.00H), 1.36–1.24 (m, 2.00H), 0.87 (t, *J* = 7.0 Hz, 1.38H), 0.81 (t, *J* = 7.3 Hz, 1.62H). ^13^C NMR (75 MHz, CDCl_3_): δ 167.0 (2), 164.2 (d, *^1^J^E^_C-F_* = 254.7 Hz), 163.3 (d, *^1^J^Z^_C-F_* = 269.0 Hz), 139.5 (d, *^3^J^E^_C-F_* = 14.3 Hz), 138.7 (d, *^3^J^Z^_C-F_* = 2.6 Hz), 129.9 (3), 129.8 (3), 128.4 (2), 128.2, 128.1, 107.8 (d, *^2^J^Z^_C-F_* = 29.7 Hz), 105.3 (d, *^2^J^E^_C-F_* = 8.5 Hz), 52.2, 52.1, 33.0 (d, *^2^J^Z^_C-F_* = 26.0 Hz), 29.0 (d, *^2^J^E^_C-F_* = 26.8 Hz), 28.5 (2), 22.4, 22.2, 13.9, 13.8. ^19^ F NMR (282 MHz, CDCl_3_): δ − 94.29 (dd, *J^E^* = 45.3, 23.4 Hz), −96.35 (dt, *J^Z^* = 38.8, 18.3 Hz). HRMS (ESI): *m*/*z* [M + H]^+^ calc. for C_14_H_18_FO_2_, 237.1285 found 237.1287.

#### 3.3.7. (*E*/*Z*)-1-(2-fluorohex-1-en-1-yl)-4-(trifluoromethyl)benzene (**3fa**)

(*E*/*Z*)-1-(2-bromo-2-fluorovinyl)-4-trifluoromethylbenzene **2f** (0.23 mmol, 62.16 mg), butyl boronic acid **2a** (0.28 mmol, 28.45 mg), Pd_2_dba_3_·CHCl_3_ (4.710^−3^ mmol, 4.25 mg), xantphos (4.710^−3^ mmol, 2.69 mg), Cs_2_CO_3_ (0.70 mmol, 227.10 mg), toluene/H_2_O (2.53 mL) were reacted according to general procedure. The crude product was purified by silica gel column chromatography (petroleum ether/EtOAc = 100:0 to 95:5) affording compound **3fa** in 95% yield (54.24 mg) as a yellow solid.^1^H NMR (300 MHz, CDCl_3_): δ 7.35–7.26 (m, 3.00H), 7.00 (d, *J* = 8.6 Hz, 1.00H), 5.93 (t, *J^E^*_H-F_ = 15.5 Hz, 0.47H), 5.22 (d, *J^Z^*_H-F_ = 38.6 Hz, 0.53H), 2.20–2.00 (m, 2.00H), 1.38–1.28 (m, 2.00H), 1.17–1.06 (m, 2.00H), 0.67 (t, *J* = 6.6 Hz, 1.41H), 0.62 (t, *J* = 6.6 Hz, 1.59H). ^13^C NMR (75 MHz, CDCl_3_): δ 164.2 (d, *^1^J^E^_C-F_* = 254.7 Hz), 163.3 (d, *^1^J^Z^_C-F_* = 268.2 Hz), 138.4 (d, *^3^J^E^_C-F_* = 15.2 Hz), 137.7 (d, *^3^J^Z^_C-F_* = 1.2 Hz), 129.7, 129.3, 128.8 (4), 128.5 (2), 128.4 (2), 125.5 (dq, *J* = 7.7, 3.8 Hz, 2), 107.4 (d, *^2^J^Z^_C-F_* = 30.0 Hz), 104.9 (d, *^2^J^E^_C-F_* = 8.5 Hz), 33.0 (d, *^2^J^Z^_C-F_* = 25.9 Hz), 29.0 (d, *^2^J^E^_C-F_* = 26.8 Hz), 28.5 (2), 22.4, 22.2, 13.9 (2). ^19^ F NMR (282 MHz, CDCl_3_): δ −62.54 (s), −94.83 (dd, *J^E^* = 44.9, 23.2 Hz), −97.08 (dt, *J^Z^* = 36.8, 18.1 Hz). HRMS (ESI): *m*/*z* [M + H]^+^ calc. for C_13_H_15_F_4_, 247.1104 found 247.1103.

#### 3.3.8. (*E*/*Z*)-1-chloro-4-(2-fluorohex-1-en-1-yl)benzene (**3ga**)

(*E*/*Z*)-1-(2-bromo-2-fluorovinyl)-4-chlorobenzene **2g** (0.23 mmol, 54.27 mg), butyl boronic acid **2a** (0.28 mmol, 28.45 mg), Pd_2_dba_3_·CHCl_3_ (4.710^−3^ mmol, 4.25 mg), xantphos (4.710^−3^ mmol, 2.69 mg), Cs_2_CO_3_ (0.70 mmol, 227.10 mg), toluene/H_2_O (2.53 mL) were reacted according to general procedure. The crude product was purified by silica gel column chromatography (petroleum ether/EtOAc = 100:0 to 95:5) affording compound **3ga** in 88% yield (43.29 mg) as a yellow solid.^1^H NMR (300 MHz, CDCl_3_): δ 7.30 (d, *J* = 8.6 Hz, 1.00H), 7.19 (dd, *J* = 11.7, 4.6 Hz, 2.00H), 7.03 (d, *J* = 8.3 Hz, 1.00H), 6.04 (d, *J^E^_H-F_* = 21.5 Hz, 0.55H), 5.34 (d, J^Z^_H-F_ = 39.0 Hz, 0.45H), 2.40–2.20 (m, 2.00H), 1.56–1.47 (m, 2.00H), 1.38–1.26 (m, 2.00H), 0.87 (t, *J* = 6.5 Hz, 1.35H), 0.81 (d, *J* = 7.3 Hz, 1.65H). ^13^C NMR (75 MHz, CDCl_3_): δ 163.3 (d, *^1^J^E^_C-F_* = 252.7 Hz), 161.9 (d, *^1^J^Z^_C-F_* = 265.9 Hz), 133.1 (d, *^3^J^E^_C-F_* = 14.3 Hz), 132.6 (d, *^3^J^Z^_C-F_* = 2.4 Hz), 132.3, 132.2, 129.9, 129.8, 129.7, 129.6, 128.7 (2), 128.6 (2), 107.3 (d, *^2^J^Z^_C-F_* = 29.5 Hz), 104.8 (d, *^2^J^E^_C-F_* = 8.8 Hz), 32.9 (d, *^2^J^Z^_C-F_* = 26.0 Hz), 28.8 (d, ^2^J^E^_C-F_ = 27.0 Hz), 28.6 (2), 22.4, 22.2, 13.9 (2). ^19^ F NMR (282 MHz, CDCl_3_): δ − 97.15 (dd, *J^E^* = 45.0, 23.2 Hz), −99.62 (dt, *J^Z^* = 39.0, 18.2 Hz). HRMS (ESI): *m*/*z* [M + H]^+^ calc. for C_12_H_15_ClF, 213.0841 found 213.0842.

#### 3.3.9. (*E*/*Z*)-1-(2-fluorohex-1-en-1-yl)-3-nitrobenzene (**3ha**)

(*E*/*Z*)-1-(2-bromo-2-fluorovinyl)-3-nitrobenzene **2h** (0.23 mmol, 56.82 mg), butyl boronic acid **2a** (0.28 mmol, 28.45 mg), Pd_2_dba_3_·CHCl_3_ (4.710^−3^ mmol, 4.25 mg), xantphos (4.710^−3^ mmol, 2.69 mg), Cs_2_CO_3_ (0.70 mmol, 227.10 mg), toluene/H_2_O (2.53 mL) were reacted according to general procedure. The crude product was purified by silica gel column chromatography (petroleum ether/EtOAc = 100:0 to 95:5) affording compound **3ha** in 99% yield (51.24 mg) as a yellow solid.^1^H NMR (300 MHz, CDCl_3_): δ 8.30 (s, 0.49H), 8.14–7.99 (m, 1.50H), 7.76 (d, *J* = 7.8 Hz, 0.51H), 7.51–7.43 (m, 1.50H), 6.22 (d, *J^E^*_H-F_ = 20.8 Hz, 0.51H), 5.55 (d, *J^Z^*_H-F_ = 37.8 Hz, 0.49H), 2.54–2.32 (m, 2.00H), 1.66–1.57 (m, 2.00H), 1.42 (dd, *J* = 15.2, 7.8 Hz, 2.00H), 0.96 (t, *J* = 6.5 Hz, 1.50H), 0.91 (t, *J* = 6.5 Hz, 1.50H). ^13^C NMR (75 MHz, CDCl_3_): δ 164.7 (d, *^1^J^E^_C-F_* = 256.0 Hz), 163.7 (d, *^1^J^Z^_C-F_* = 269.1 Hz), 148.6 (2), 136.4 (d, *^3^J^E^_C-F_* = 14.9 Hz), 135.7 (d, *^3^J^Z^_C-F_* = 2.2 Hz), 134.5 (d, *J_C-F_* = 2.7 Hz), 134.1 (d, *J_C-F_* = 7.9 Hz), 129.5, 129.3, 123.2 (d, *J_C-F_* = 2.8 Hz), 123.0 (d, *J_C-F_* = 8.1 Hz), 121.6, 121.4 (d, *J_C-F_* = 1.9 Hz), 106.7 (d, *^2^J^Z^_C-F_* = 31.3 Hz), 104.2 (d, *^2^J^E^_C-F_* = 8.4 Hz), 32.9 (d, *^2^J^Z^_C-F_* = 25.8 Hz), 28.9 (d, *^2^J^E^_C-F_* = 26.9 Hz), 28.4 (2), 22.4, 22.2, 13.9 (2). ^19^ F NMR (282 MHz, CDCl_3_): δ −93.83 (td, *J^E^* = 23.2, 20.9 Hz), −96.00–−96.53 (m). HRMS (ESI): *m*/*z* [M + H]^+^ calc. for C_12_H_15_FNO_2_, 224.1081 found 224.1079.

#### 3.3.10. (*E*/*Z*)-1-(2-fluorohex-1-en-1-yl)-2-methoxybenzene (**3ia**)

(*E*/*Z*)-1-(2-bromo-2-fluorovinyl)-2-methoxybenzene **2i** (0.23 mmol, 53.35 mg), butyl boronic acid **2a** (0.28 mmol, 28.45 mg), Pd_2_dba_3_·CHCl_3_ (4.710^−3^ mmol, 4.25 mg), xantphos (4.710^−3^ mmol, 2.69 mg), Cs_2_CO_3_ (0.70 mmol, 227.10 mg), toluene/H_2_O (2.53 mL) were reacted according to general procedure. The crude product was purified by silica gel column chromatography (petroleum ether/EtOAc = 100:0 to 95:5) affording compound **3ia** in 81% yield (39.11 mg) as a yellow solid.^1^H NMR (300 MHz, CDCl_3_): ^1^H NMR (300 MHz, CDCl_3_) δ 7.67 (dt, *J* = 8.2, 4.1 Hz, 0.41H), 7.18–7.05 (m, 1.59H), 6.86–6.71 (m, 2.00H), 6.15 (d, *J^E^_H-F_* = 21.9 Hz, 0.59H), 5.79 (d, *J^Z^_H-F_* = 40.6 Hz, 0.41H), 3.74 (s, 3.00H), 2.38–2.23 (m, 2.00H), 1.53 (ddd, *J* = 8.2, 7.2, 5.2 Hz, 2.00H), 1.31 (dd, *J* = 14.9, 7.6 Hz, 2.00H), 0.87 (t, *J* = 7.3 Hz, 1.23H), 0.81 (t, *J* = 7.3 Hz, 1.77H). ^13^C NMR (75 MHz, CDCl_3_): δ 162.6 (d, *^1^J^E^_C-F_* = 250.6 Hz), 161.4 (d, *^1^J^Z^_C-F_* = 264.4 Hz), 157.2 (d, *J_C-F_* = 2.7 Hz), 156.0, 129.9 (d, *J_C-F_* = 12.7 Hz), 129.7 (d, *J_C-F_* = 1.6 Hz), 128.3, 127.8 (d, *J_C-F_* = 1.7 Hz), 123.5 (d, *^3^J^E^_C-F_* = 13.8 Hz), 122.8 (d, *^3^J^Z^_C-F_* = 2.7 Hz), 120.7, 120.5, 110.6 (2), 103.7 (d, *^2^J^Z^_C-F_* = 30.4 Hz), 99.1 (d, *^2^J^E^_C-F_* = 7.4 Hz), 55.7, 55.6, 33.2 (d, *^2^J^Z^_C-F_* = 26.7 Hz), 28.9 (d, *^2^J^E^_C-F_* = 27.2 Hz), 28.7 (2), 22.5, 22.2, 13.9 (2). ^19^ F NMR (282 MHz, CDCl_3_): δ − 98.74 (q, *J^E^* = 22.7 Hz), −102.28 (dt, *J^Z^* = 40.6, 18.0 Hz). HRMS (ESI): *m*/*z* [M + H]^+^ calc. for C_13_H_18_FO, 209.1336 found 209.1333.

#### 3.3.11. (2-Bromo-2-fluoroethene-1,1-diyl)dibenzene (**3ja**)

1-(2-bromo-2-fluorovinyl)-2-methoxybenzene **2j** (0.23 mmol, 64.03 mg), butyl boronic acid **2a** (0.28 mmol, 28.45 mg), Pd_2_dba_3_·CHCl_3_ (4.710^−3^ mmol, 4.25 mg), xantphos (4.710^−3^ mmol, 2.69 mg), Cs_2_CO_3_ (0.70 mmol, 227.10 mg), toluene/H_2_O (2.53 mL) were reacted according to general procedure. The crude product was purified by silica gel column chromatography (petroleum ether/EtOAc = 100:0 to 95:5) affording compound **3ja** in 90% yield (53.06 mg) as a yellow solid.^1^H NMR (300 MHz, CDCl_3_): δ 7.39–7.13 (m, 10H), 2.40–2.21 (m, 2H), 1.64–1.52 (m, 2H), 1.31 (dt, *J* = 14.6, 7.4 Hz, 2H), 0.85 (t, *J* = 7.3 Hz, 3H). ^13^C NMR (75 MHz, CDCl_3_): δ 158.5 (d, *^1^J^E^_C-F_* = 261.1 Hz), 139.3 (d, *^3^J_C-F_* = 8.3 Hz), 137.9, 130.4 (d, *J_C-F_* = 2.6 Hz), 129.7 (d, *J_C-F_* = 4.9 Hz), 128.5 (3), 128.1 (3), 127.2, 126.8, 120.4 (d, *^2^J_C-F_* = 15.2 Hz), 30.3 (d, *^2^J_C-F_* = 27.4 Hz), 29.0, 22.3, 13.9. ^19^ F NMR (282 MHz, CDCl_3_): δ − 106.14 (t, *J* = 23.0 Hz). HRMS (ESI): *m*/*z* [M + H]^+^ calc. for C_18_H_20_F, 255.1543 found 255.1541.

#### 3.3.12. (E/Z)-1-(3-fluorohept-2-en-2-yl)-4-methoxybenzene (**3ka**)

(*E*/*Z*)-1-(1-bromo-1-fluoroprop-1-en-2-yl)-4-methoxybenzene **2k** (0.23 mmol, 56.60 mg), butyl boronic acid **2a** (0.28 mmol, 28.45 mg), Pd_2_dba_3_·CHCl_3_ (4.710^−3^ mmol, 4.25 mg), xantphos (4.710^−3^ mmol, 2.69 mg), Cs_2_CO_3_ (0.70 mmol, 227.10 mg), toluene/H_2_O (2.53 mL) were reacted according to general procedure. The crude product was purified by silica gel column chromatography (petroleum ether/EtOAc = 100:0 to 95:5) affording compound **3ka** in 90% yield (43.80 mg) as a yellow solid.^1^H NMR (300 MHz, CDCl_3_): δ 7.31 (dd, *J* = 8.9, 1.1 Hz, 1.0H), 7.11 (d, *J* = 8.6 Hz, 1.0H), 6.87 (dd, *J* = 8.9, 2.2 Hz, 2.0H), 3.82 (s, 1.5H), 3.81 (s, 1.5H), 2.40 (dt, *J* = 24.0, 7.3 Hz, 1.0H), 2.28–2.07 (m, 1.0H), 2.07–1.81 (m, 3.0H), 1.60–1.26 (m, 4.0H), 0.96 (t, *J* = 7.2 Hz, 1.5H), 0.85 (t, *J* = 7.3 Hz, 1.5H). ^13^C NMR (75 MHz, CDCl_3_): 158.5, 158.3, 157.1 (d, *^1^J^E^_C-F_* = 248.6 Hz), 155.6 (d, *^1^J^E^_C-F_* = 248.7 Hz), 133.0 (d, *^3^J^E^_C-F_* = 9.3 Hz), 131.2, 129.6 (d, *J_C-F_* = 2.7 Hz, 2), 129.4 (d, *J_C-F_* = 4.2 Hz, 2), 113.8 (2), 113.5 (2), 111.3, (d, *^2^J_C-F_* = 13.8 Hz, 2), 55.4 (2), 29.3 (d, *J_C-F_* = 19.6 Hz), 29.0 (d, *J* = 2.9 Hz), 29.0, 28.9, 22.3 (2), 17.4 (d, *^3^J^Z^_C-F_* = 4.7 Hz), 16.4 (d, *^3^J^E^_C-F_* = 7.9 Hz), 14.0, 13.9. ^19^ F NMR (282 MHz, CDCl_3_): δ −107.87–−108.18 (m), −109.64 (ddd, *J* = 26.7, 19.6, 3.6 Hz). HRMS (ESI): *m*/*z* [M + H]^+^ calc. for C_14_H_20_FO, 223.1492 found 223.1490.

#### 3.3.13. 1-(2-Fluoroprop-1-en-1-yl)-4-nitrobenzene (**3ab**)

(*E*/*Z*)-1-(2-bromo-2-fluorovinyl)-4-nitrobenzene **1a** (0.23 mmol, 56.32 mg), methyl boronic acid **2b** (0.28 mmol, 16.71 mg), Pd_2_dba_3_·CHCl_3_ (4.710^−3^ mmol, 4.25 mg), xantphos (4.710^−3^ mmol, 2.69 mg), Cs_2_CO_3_ (0.70 mmol, 227.10 mg), toluene/H_2_O (2.53 mL) were reacted according to general procedure. The crude product was purified by silica gel column chromatography (petroleum ether/EtOAc = 100:0 to 95:5) affording compound **3ab** in 86% yield (36.12 mg) as a yellow solid.^1^H NMR (300 MHz, CDCl_3_): δ 8.26–8.11 (m, 2.00H), 7.56 (t, *J* = 8.5 Hz, 1.26H), 7.35 (d, *J* = 8.6 Hz, 0.76H), 6.25 (d, *J* = 20.7 Hz, 0.37H), 5.58 (d, *J* = 37.4 Hz, 0.63H), 2.22–2.09 (m, 3.00H). ^13^C NMR (75 MHz, CDCl_3_): δ 164.1 (d, *^1^J^E^_C-F_* = 257.5 Hz), 163.6 (d, *^1^J^Z^_C-F_* = 271.8 Hz), 145.3 (2), 140.5 (d, *^3^J^E^_C-F_* = 14.8 Hz), 139.6 (d, *^3^J^Z^_C-F_* = 2.7 Hz), 129.2 (2), 129.1, 129.0, 124.2 (2), 123.9 (2), 111.8 (d, *^2^J^Z^_C-F_* = 6.0 Hz), 110.7 (d, *^2^J^E^_C-F_* = 26.1 Hz), 19.40 (d, *^2^J^Z^_C-F_* = 2.5 Hz), 18.9 (d, *^2^J^E^_C-F_* = 24.9 Hz). ^19^ F NMR (282 MHz, CDCl_3_): δ − 81.75 (tt, *J* = 36.1, 18.0 Hz), −87.75 (dq, *J* = 37.5, 17.1 Hz). HRMS (ESI): *m*/*z* [M + H]^+^ calc. for C_9_H_8_FNO_2_, 182.0612 found 182.0619.

#### 3.3.14. 1-(2-Fluoropent-1-en-1-yl)-4-nitrobenzene (**3ac**)

(*E*/*Z*)-1-(2-bromo-2-fluorovinyl)-4-nitrobenzene **1a** (0.23 mmol, 56.32 mg), pentyl boronic acid **2c** (0.28 mmol, 24.51 mg), Pd_2_dba_3_·CHCl_3_ (4.710^−3^ mmol, 4.25 mg), xantphos (4.710^−3^ mmol, 2.69 mg), Cs_2_CO_3_ (0.70 mmol, 227.10 mg), toluene/H_2_O (2.53 mL) were reacted according to general procedure. The crude product was purified by silica gel column chromatography (petroleum ether/EtOAc = 100:0 to 95:5) affording compound **3ac** in 95% yield (46.08 mg) as a yellow solid.^1^H NMR (300 MHz, CDCl_3_): δ 8.24–8.12 (m, 2.00H), 7.62–7.56 (m, 1.24H), 7.36–7.31 (m, 0.76H), 6.24 (d, *J^E^*_H-F_ = 21.1 Hz, 0.38H), 5.57 (d, *J^Z^*_H-F_ = 38.1 Hz, 0.62H), 2.51–2.30 (m, 2.00H), 1.67 (ddd, *J* = 14.8, 7.4, 5.7 Hz, 2.00H), 1.00 (dd, *J* = 15.7, 7.7 Hz, 3.00H). ^13^C NMR (75 MHz, CDCl_3_): δ 164.1 (d, *^1^J^E^_C-F_* = 257.5 Hz), 163.6 (d, *^1^J^Z^_C-F_* = 271.8 Hz), 143.5 (2), 139.7 (d, *^3^J^E^_C-F_* = 14.9 Hz), 138.8 (d, *^3^J^Z^_C-F_* = 2.5 Hz), 127.1 (2), 126.9, 126.8, 121.9 (4), 105.5 (d, *^2^J^Z^_C-F_* = 31.1 Hz), 102.8 (d, *^2^J^E^_C-F_* = 8.1 Hz), 33.3 (d, *^2^J^Z^_C-F_* = 25.7 Hz), 29.3 (d, *^2^J^E^_C-F_* = 26.8 Hz), 17.7 (2), 11.7, 11.5. ^19^ F NMR (282 MHz, CDCl_3_): δ − 91.24 (dd, *J^E^* = 44.6, 23.2 Hz), −93.61–−94.11 (m). HRMS (ESI): *m*/*z* [M + H]^+^ calc. for C_11_H_13_FNO_2_, 210.0925 found 210.0920.

#### 3.3.15. 1-(2-Fluoro-4-phenylbut-1-en-1-yl)-4-nitrobenzene (**3ad**)

(*E*/*Z*)-1-(2-bromo-2-fluorovinyl)-4-nitrobenzene **1a** (0.23 mmol, 56.32 mg), phenethylboronic acid **2d** (0.28 mmol, 41.78 mg), Pd_2_dba_3_·CHCl_3_ (4.710^−3^ mmol, 4.25 mg), xantphos (4.710^−3^ mmol, 2.69 mg), Cs_2_CO_3_ (0.70 mmol, 227.10 mg), toluene/H_2_O (2.53 mL) were reacted according to general procedure. The crude product was purified by silica gel column chromatography (petroleum ether/EtOAc = 100:0 to 95:5) affording compound **3ad** in 87% yield (54.71 mg) as a yellow solid.^1^H NMR (300 MHz, CDCl_3_): δ 8.16 (d, *J* = 9.0 Hz, 1.24H), 8.08 (d, *J^E^*_H-F_ = 8.9 Hz, 0.76H), 7.57 (d, *J^Z^*_H-F_ = 8.9 Hz, 1.24H), 7.35–7.15 (m, 5.00H), 7.07–7.00 (m, 0.76H), 6.25 (d, *J^E^*_H-F_ = 20.9 Hz, 0.38H), 5.53 (d, *J^Z^*_H-F_ = 38.0 Hz, 0.62H), 2.95 (dd, *J* = 8.8, 6.9 Hz, 2.00H), 2.70 (ddd, *J* = 16.2, 10.9, 7.4 Hz, 2.00H). ^13^C NMR (75 MHz, CDCl_3_): δ 163.4 (d, *^1^J^E^_C-F_* = 261.0 Hz), 163.2 (d, *^1^J^Z^_C-F_* = 266.8 Hz), 146.2, 146.1, 141.1, 140.9, 140.3 (d, *^3^J^Z^_C-F_* = 2.4 Hz), 140.0 (d, *^3^J^E^_C-F_* = 10.6 Hz), 129.0, 128.9, 128.8, 128.7 (2), 128.6 (4), 128.5, 128.4 (2), 126.6, 126.5, 123.7 (2), 123.7 (2), 108.1 (d, *^2^J^Z^_C-F_* = 30.3 Hz), 105.3 (d, *^2^J^E^_C-F_* = 7.9 Hz), 35.2 (d, *^2^J^Z^_C-F_* = 25.8 Hz), 32.3 (d, *^2^J^E^_C-F_* = 36.5 Hz), 31.5, 31.2. ^19^ F NMR (282 MHz, CDCl_3_): δ − 93.83 (dd, *J^E^* = 43.3, 22.6 Hz), −95.15 (dt, *J^Z^* = 36.5, 18.0 Hz). HRMS (ESI): *m*/*z* [M + H]^+^ calc. for C_16_H_15_FNO_2_, 272.1081 found 272.1082.

#### 3.3.16. 1-(2-Fluoro-4-methylpent-1-en-1-yl)-4-nitrobenzene (**3ae**)

(*E*/*Z*)-1-(2-bromo-2-fluorovinyl)-4-nitrobenzene **1a** (0.23 mmol, 56.32 mg), isobutylboronic acid **2e** (0.28 mmol, 28.42 mg), Pd_2_dba_3_·CHCl_3_ (4.710^−3^ mmol, 4.25 mg), xantphos (4.710^−3^ mmol, 2.69 mg), Cs_2_CO_3_ (0.70 mmol, 227.10 mg), toluene/H_2_O (2.53 mL) were reacted according to general procedure. The crude product was purified by silica gel column chromatography (petroleum ether/EtOAc = 100:0 to 95:5) affording compound **3ae** in 77% yield (39.85 mg) as a yellow solid.^1^H NMR (300 MHz, CDCl_3_): δ 8.18 (d, *J* = 7.4 Hz, 0.84H), 8.15 (d, *J* = 7.5 Hz, 1.16H), 7.59 (d, *J* = 9.0 Hz, 1.16H), 7.34 (d, *J* = 8.4 Hz, 0.84H), 6.27 (d, *J^E^*_H-F_ = 21.6 Hz, 0.42H), 5.56 (d, *J^Z^*_H-F_ = 37.9 Hz, 0.58H), 2.35 (dd, *J* = 23.4, 7.2 Hz, 0.84H), 2.23 (dd, *J* = 21.4, 7.1 Hz, 1.16H), 2.08–1.95 (m, 1.00H), 1.00 (dd, *J* = 6.6, 0.5 Hz, 3.48H), 0.96 (dd, *J* = 6.7, 0.6 Hz, 2.52H). ^13^C NMR (75 MHz, CDCl_3_): 164.3 (d, *^1^J^E^_C-F_* = 257.5 Hz), 163.8 (d, *^1^J^Z^_C-F_* = 272.2 Hz), 141.5 (d, *^3^J^E^_C-F_* = 15.1 Hz), 140.6 (d, *^3^J^Z^_C-F_* = 2.6 Hz), 129.1 (d, *J_C-F_* = 2.7 Hz, 2), 128.7 (d, *J_C-F_* = 8.3 Hz, 2), 123.8 (4), 108.0 (d, *^2^J^Z^_C-F_* = 31.1 Hz), 105.7 (d, *^2^J^E^_C-F_* = 8.3 Hz), 42.5 (d, *^2^J^E^_C-F_* = 25.0 Hz), 38.0 (d, *^2^J^Z^_C-F_* = 25.9 Hz), 26.2, 26.1, 22.3, 22.2. ^19^ F NMR (282 MHz, CDCl_3_): δ − 89.63 (q, *J^E^* = 22.9 Hz), −93.10 (dt, *J^Z^* = 37.9, 21.4 Hz). HRMS (ESI): *m*/*z* [M + H]^+^ calc. for C_12_H_15_FNO_2_, 224.1081 found 224.1080.

#### 3.3.17. 1-(2-Cyclopropyl-2-fluorovinyl)-4-nitrobenzene (**3ah**)

(*E*/*Z*)-1-(2-bromo-2-fluorovinyl)-4-nitrobenzene **1a** (0.23 mmol, 56.32 mg), cyclopropylboronic acid **2h** (0.28 mmol, 23.95 mg), Pd_2_dba_3_·CHCl_3_ (4.710^−3^ mmol, 4.25 mg), xantphos (4.710^−3^ mmol, 2.69 mg), Cs_2_CO_3_ (0.70 mmol, 227.10 mg), toluene/H_2_O (2.53 mL) were reacted according to general procedure. The crude product was purified by silica gel column chromatography (petroleum ether/EtOAc = 100:0 to 95:5) affording compound **3ah** in 83% yield (39.87 mg) as a yellow solid.^1^H NMR (300 MHz, CDCl_3_): δ 8.12 (d, *J* = 8.9 Hz, 0.84H), 8.07 (d, *J* = 9.0 Hz, 1.16H), 7.47 (d, *J* = 9.0 Hz, 1.16H), 7.41 (d, *J* = 8.4 Hz, 0.84H), 6.16 (d, *J^E^*_H-F_ = 19.9 Hz, 0.42H), 5.58 (d, *J^Z^*_H-F_ = 37.9 Hz, 0.58H), 1.93–1.77 (v-1Hm, 0.42H), 1.69–1.54 (m, 0.58H), 0.92 (dt, *J* = 8.9, 3.2 Hz, 0.84H), 0.87–0.78 (m, 3.16H). ^13^C NMR (75 MHz, CDCl_3_): 164.2 (d, *^1^J^Z^_C-F_* = 266.7 Hz), 164.0 (d, *^1^J^E^_C-F_* = 252.9 Hz), 146.1, 147.5, 141.8 (d, *^3^J^E^_C-F_* = 14.7 Hz), 140.8 (d, *^3^J^Z^_C-F_* = 3.3 Hz), 129.1 (d, *J_C-F_* = 2.7 Hz, 2), 128.4 (d, *J_C-F_* = 8.2 Hz, 2), 123.(4),.106.2 (d, *^2^J^Z^_C-F_* = 32.9 Hz), 102.9 (d, *^2^J^E^_C-F_* = 9.9 Hz), 13.4 (d, *^2^J^Z^_C-F_* = 28.1 Hz), 10.3 (d, *^2^J^E^_C-F_* = 26.6 Hz), 6.2, 6.1, 5.8 (2). ^19^ F NMR (282 MHz, CDCl_3_): δ − 108.50 (dd, *J* = 37.9, 22.7 Hz). HRMS (ESI): *m*/*z* [M + H]^+^ calc. for C_11_H_11_FNO_2_, 208.0768 found 208.0765.

## 4. Conclusions

In conclusion, an efficient palladium-catalyzed carbon–carbon coupling reaction of readily available *gem*-bromofluoroalkenes with primary and strained secondary alkyl boronic acid derivatives was successfully achieved under mild conditions. This methodology demonstrates its applicability for the synthesis of alkyl trisubstituted or tetrasubstituted monofluoroalkenes with a broad range of *gem*-bromofluoroalkenes and alkyl boronic acids with good group compatibility, stereospecificity and excellent yields. Such reactions may be useful for the synthesis of fluoroolefins of interest for life and material sciences.

## Figures and Tables

**Table 1:** Optimization of the cross-coupling between *E*/*Z*-**1a** and butylboronic acid **2a** ^a^.

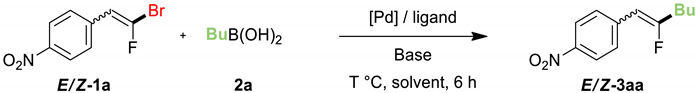								
Entry	*E*/*Z* 1a ^b^	[Pd]	Ligand	Base	Solvent	T (°C)	*Z*/*E* 3aa ^b^	Yield (%) ^c^
1	55:45	Pd(Ph_3_)_4_	-	Cs_2_CO_3_	toluene/H_2_O	80	-	-
2	55:45	Pd_2_dba_3_ CHCl_3_	-	Cs_2_CO_3_	toluene/H_2_O	80	-	-
3	56:44	PdCl_2_dppf	-	Cs_2_CO_3_	toluene/H_2_O	80	58:42	95
4	55:45	PdCl_2_dppf	-	K_2_CO_3_	toluene/H_2_O	80	46:54	76
5	55:45	PdCl_2_dppf	-	Na_2_CO_3_	toluene/H_2_O	80	57:43	44
6	55:45	PdCl_2_dppf	-	K_3_PO_4_	toluene/H_2_O	80	51:49	93
7	55:46	PdCl_2_dppf	-	Ba(OH)_2_	toluene/H_2_O	80	-	-
8	39:61	PdCl_2_dppf	-	Cs_2_CO_3_	toluene	80	38:62	89
9	39:61	PdCl_2_dppf	-	Cs_2_CO_3_	Dioxane	80	-	-
10	39:61	PdCl_2_dppf	-	Cs_2_CO_3_	THF	80	-	-
11	55:45	PdCl_2_dppf	-	Cs_2_CO_3_	toluene/H_2_O	RT	-	-
12	55:45	PdCl_2_dppf	-	Cs_2_CO_3_	toluene/H_2_O	60	45:55	51
13	55:45	Pd_2_dba_3._CHCl_3_	Dppf	Cs_2_CO_3_	toluene/H_2_O	80	51:49	91
14	48:52	Pd_2_dba_3_ CHCl_3_	Dppe	Cs_2_CO_3_	toluene/H_2_O	80	48:52	41
15	48:52	Pd_2_dba_3_ CHCl_3_	Xantphos	Cs_2_CO_3_	toluene/H_2_O	80	49:51	99
16	48:52	Pd_2_dba_3_ CHCl_3_	Xphos	Cs_2_CO_3_	toluene/H_2_O	80	60:40	35
17	48:52	Pd_2_dba_3_ CHCl_3_	TFP	Cs_2_CO_3_	toluene/H_2_O	80	42:58	78

^a^ All reactions unless specified were carried out using **1a** (1 eq), **2a** (1.2 eq), Pd source (2 mol%), ligand (2 mol%) and base (3 eq), in solvent (0.09 M) under N_2_ for 6 h. ^b^ Ratio determined by ^1^H NMR. ^c^ Yield was determined using 1,3,5-trimethoxybenzene as an internal standard. Pd_2_dba_3_ = tris(dibenzylideneacetone)dipalladium. Dppf = 1,1′-bis(diphenylphosphino)ferrocene. Dppe = 1,2-bis(diphenylphosphino)ethane. Xantphos = 4,5-bis(diphenylphosphino)-9,9-dimethylxanthene. Xphos = 2-dicyclohexylphosphino-2′,4′,6′-triisopropylbiphenyl. TFP = tri(2-furyl)phosphine.

## Supplementary Materials

^1^H-, ^13^C- and ^19^F-NMR spectra of products associated with this article are available online.
