# Supplementary material for: Palladium-Catalyzed Cross-Coupling of Gem-Bromofluoroalkenes with Alkylboronic Acids for the Synthesis of Alkylated Monofluoroalkenes
Source: Molecules. 2020 Nov 25;25(23):5532. doi: 10.3390/molecules25235532 (PMC7728317; doi:10.3390/molecules25235532)

Supporting Information

Palladium-Catalyzed Cross-Coupling of *Gem*-Bromofluoroalkenes with Alkylboronic Acids for the Synthesis of Alkylated Monofluoroalkenes

Laëtitia Chausset-Boissarie *, Nicolas Cheval and Christian Rolando *

Univ. Lille, CNRS, USR 3290—MSAP—Miniaturisation pour la Synthèse, l'Analyse et la Protéomique, F-59000 Lille, France; nicolas.cheval@gmail.fr

* Correspondence: laetitia.boissarie@univ-lille.fr (L.C.-B.); christian.rolando@univ-lille.fr (C.R.)

Academic Editors: Vito Capriati and Yurii Yagupolskii

Received: 15 October 2020; Accepted: 19 November 2020; Published: 25 November 2020

I. MATERIALS AND METHODS 2

II. EXPERIMENTAL PROCEDURES AND CHARACTERIZATION DATA 3

A. Synthesis of *Gem*-bromofluoroalkenes. 3

B. General procedure for cross-coupling reaction 3

III. COPIES OF NMR AND HRMS SPECTRA 4

I. Materials and methods

All reagents were purchased from commercial suppliers (Strem Chemicals Inc., Sigma-Aldrich or Alfa Aesar) and were used without further purification unless otherwise indicated. Non commercially available substrate were synthesized following protocols specified in Sections A.

Thin-layer chromatography (TLC) were performed on Silica gel 60 F254 plates (Merck) and visualized under UV (254 nm) or by staining with potassium permanganate or phosphomolybdic acid.

The purification of the obtained products was performed by flash chromatography using PuriFlash® 215 equipped with UV 1 (90–840 nm) and ELSD detectors purchased from Interchim. Pre-packed flash chromatography Silica HP columns (30 μm) were used.

NMR spectra were recorded on a Bruker AVANCE 300 spectrometer at 300 MHz (75 MHz). Chemical shifts are given in parts per million relative to solvent signal. The following abbreviations are used for the proton spectra multiplicities: s, singlet; d, doublet; t, triplet; q, quartet; m, multiplet. Coupling constant are reported in hertz (Hz).

UV-Vis spectra were recorded on a UV-Vis spectrophotometer (Lambda 25, Perking Elmer).

Fluorescence Flow-through cell (176.751-QS, optical path length 3 × 3 mm, V 100 μL, Center Height Z 8.5 mm, Hellma Analytics) was used for all the UV-Vis measurements.

High-resolution mass spectra (HRMS) were performed on a ThermoFisher Scientific LTQ Orbitrap XL mass spectrometer using electrospray ionization (ESI).

II. Experimental procedures and characterization data

A. Synthesis of *Gem*-bromofluoroalkenes.

Gem-bromofluoroalkenes **1a-o** were synthetized according to known procedures from the appropriate aldehyde and tribromofluoromethane.[1]

B. General procedure for cross-coupling reaction

In a slenck tube was added gem-bromofluoroalkene **1** (1.0 equiv), boronic acid (1.2 equiv), $Pd_2dba_3.CHCl_3$ (2 mol %), Xantphos (2 mol %) and Cs_2CO_3 (3 equiv). The vial was flushed under nitrogen, then filled with a mixture of Toluene/H_2O ([0.09]). The reaction mixture was heated during 6 h at 80 °C then cooled to r.t., filtered through Celite and washed with EtOAc. The filtrate was concentrated under vacuum and the residue was purified by flash chromatography on silica gel (Petroleum ether/EtOAc = 100:0 to 95:5) to afford the pure product **3**.

[1] Lei, X.; Dutheuil, G.; Pannecoucke, X.; Quirion, J-C, *Org. Lett.* **2004**, *6*, 2101

III. Copies of NMR and HRMS spectra

(*E*/*Z*)-1-(2-fluorohex-1-en-1-yl)-4-nitrobenzene 3aa

^{1}H NMR Spectrum ($CDCl_3$, 300 MHz)

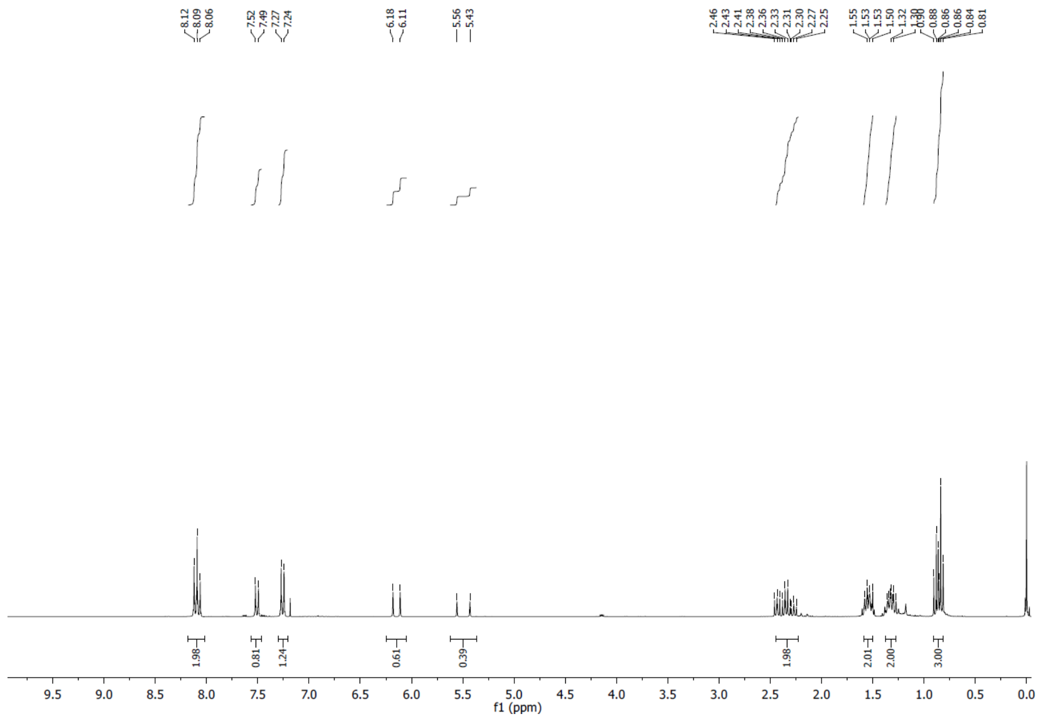

^{13}C ($CDCl_3$, 75 MHz)

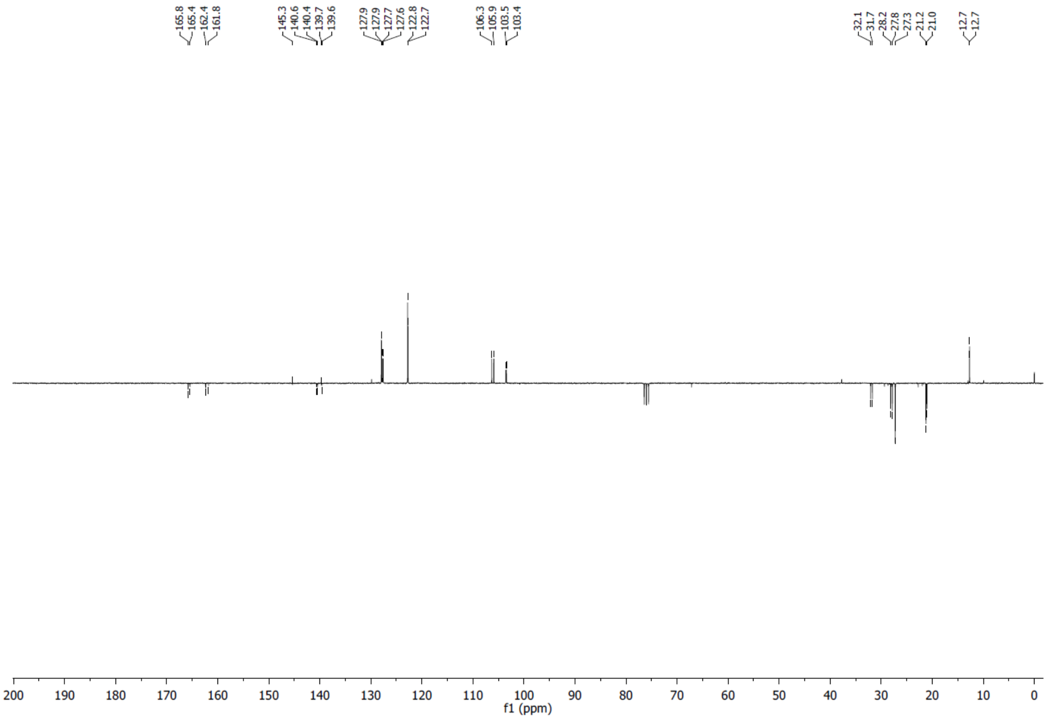

^{19}F NMR (282.5 MHz, $CDCl_3$)

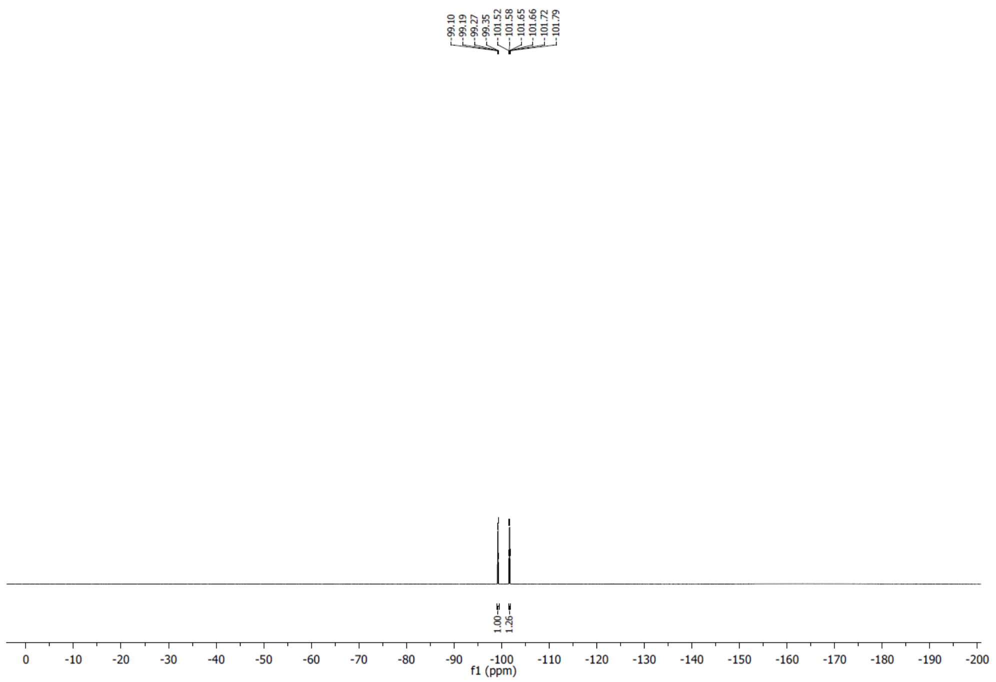

HRMS-spectrum

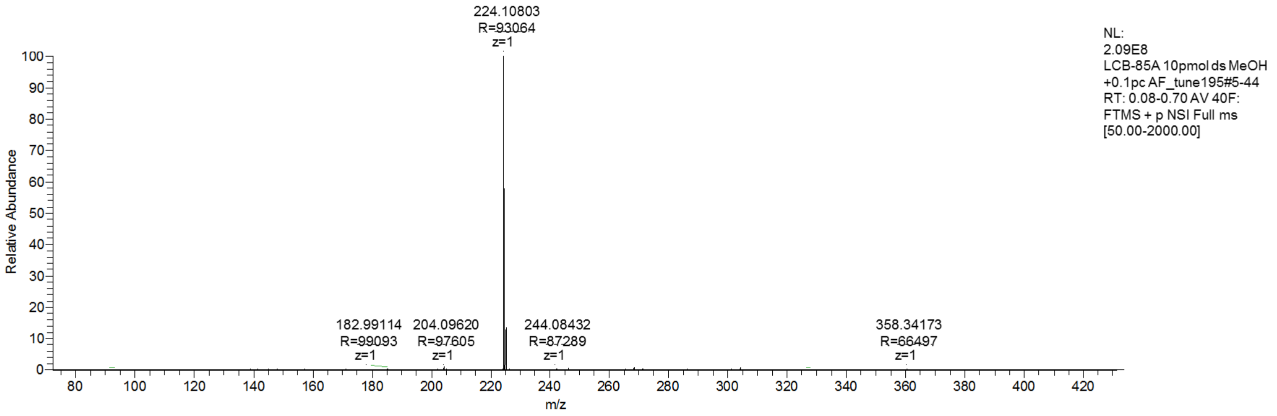

(*E*/*Z*)-(2-fluorohex-1-en-1-yl)benzene 3ba

^1H NMR Spectrum ($CDCl_3$, 300 MHz)

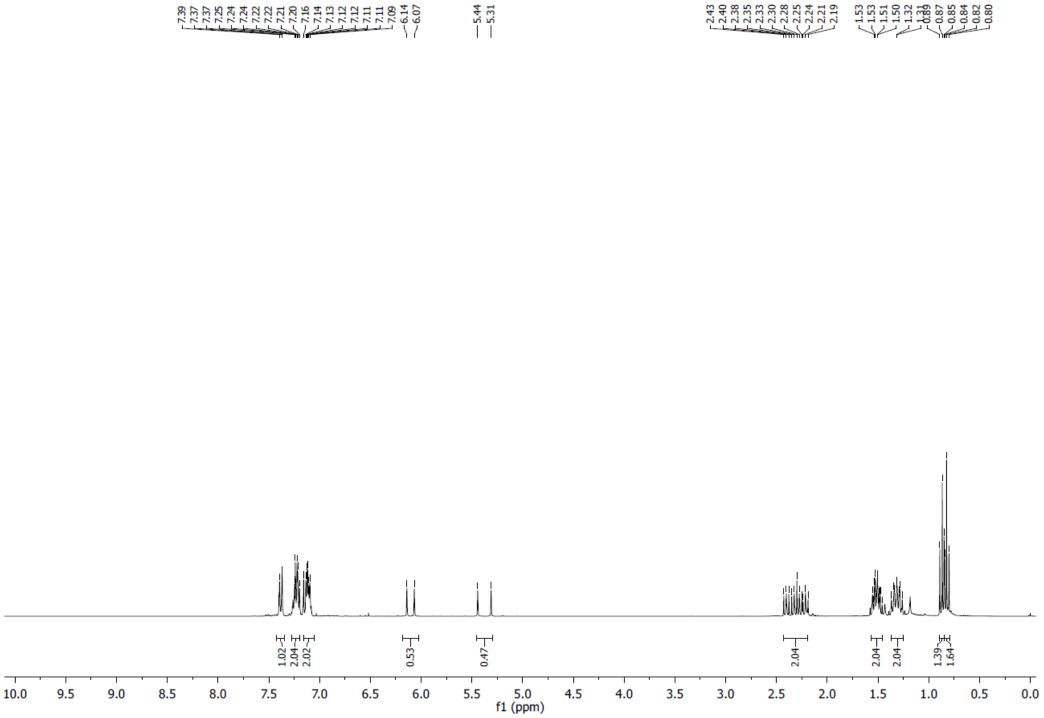

^{13}C ($CDCl_3$, 75 MHz)

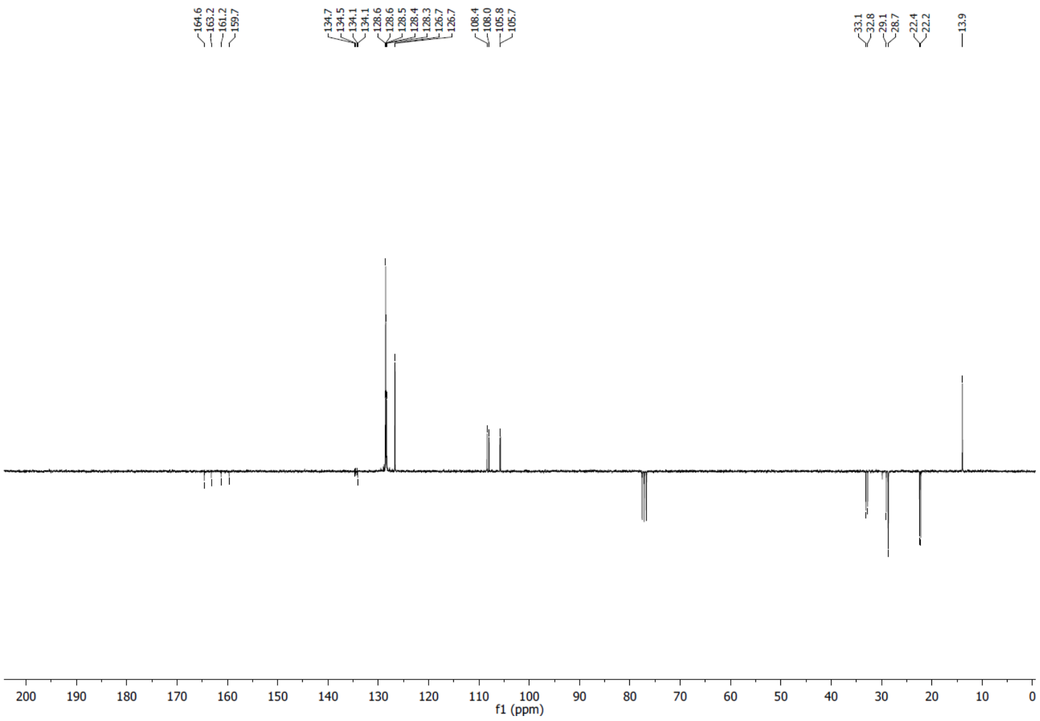

^{19}F NMR (282.5 MHz, CDCl$_3$)

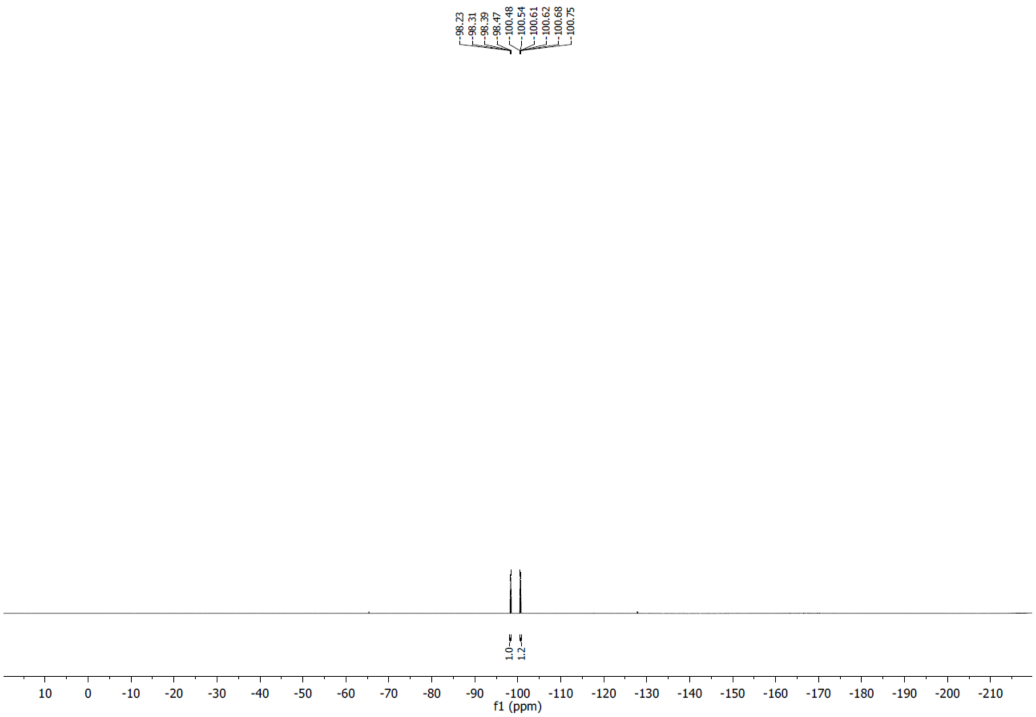

HRMS-spectrum

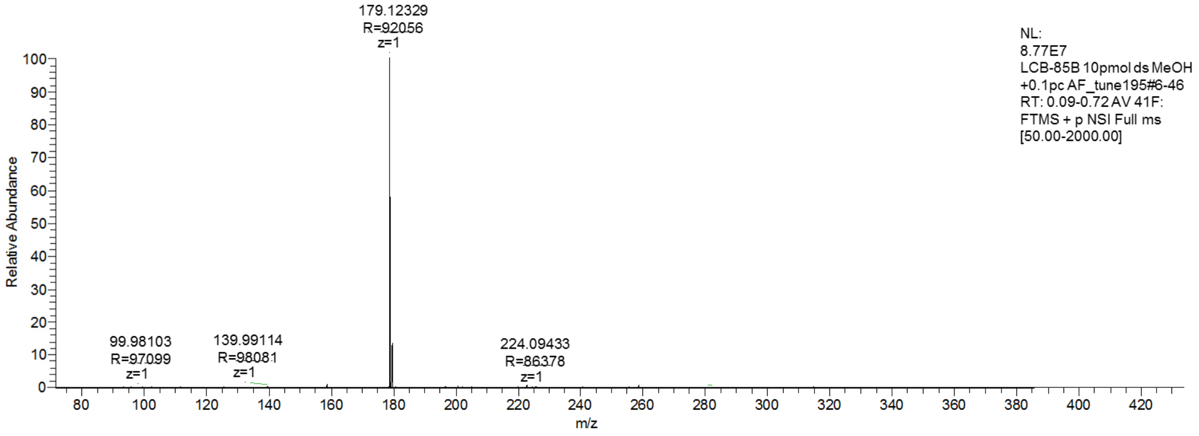

(*E*/*Z*)-1-(2-fluorohex-1-en-1-yl)-4-methylbenzene 3ca

^{1}H NMR Spectrum ($CDCl_3$, 300 MHz)

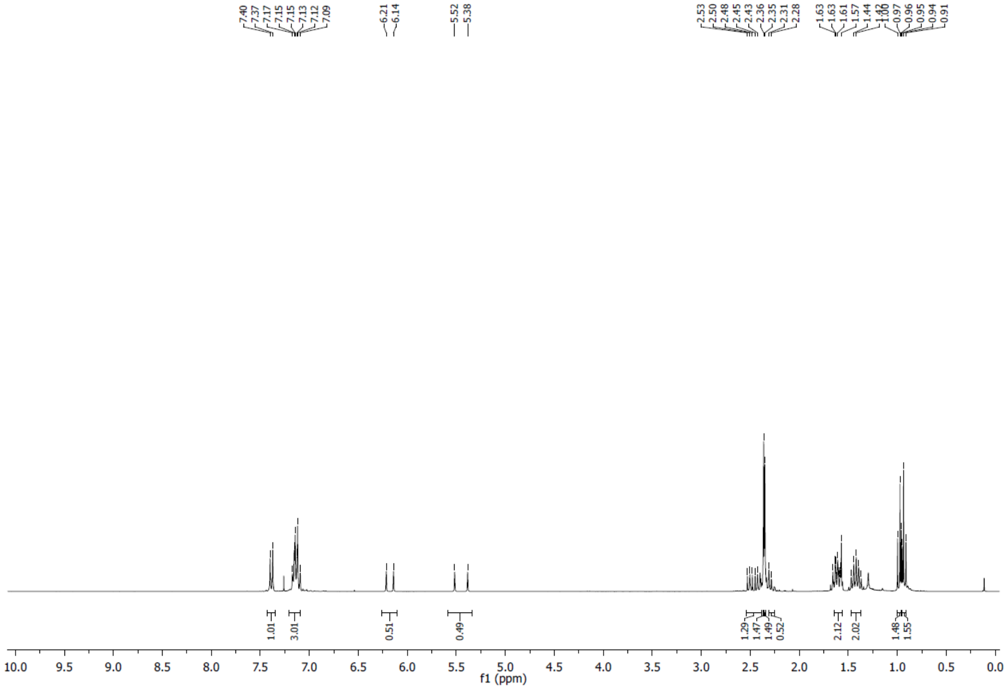

^{13}C ($CDCl_3$, 75 MHz)

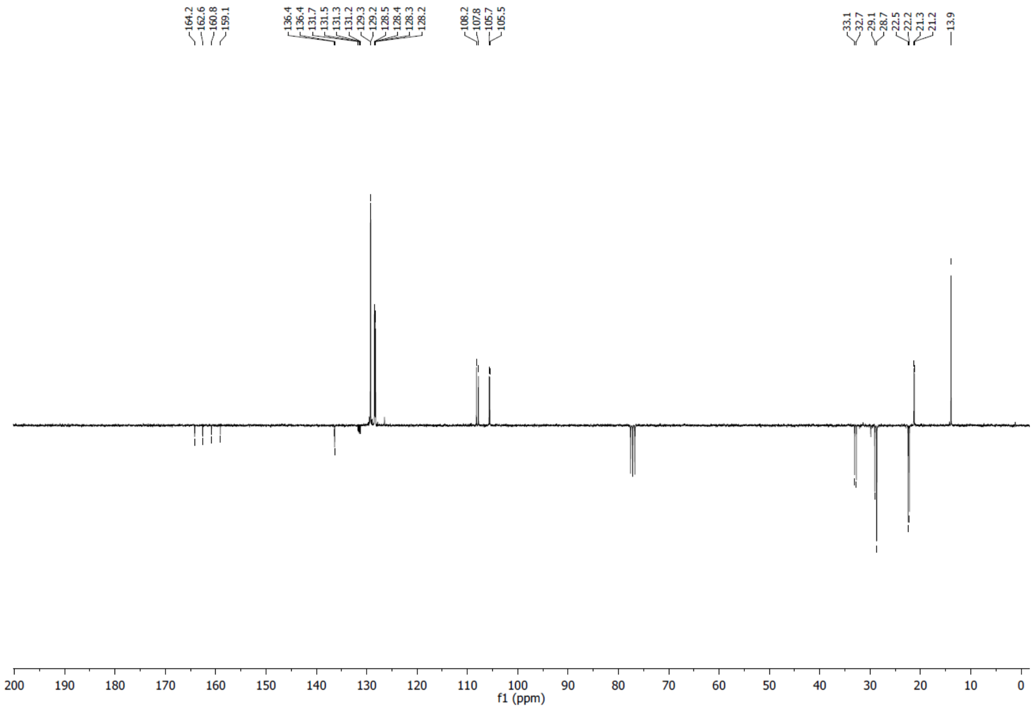

^{19}F NMR (282.5 MHz, $CDCl_3$)

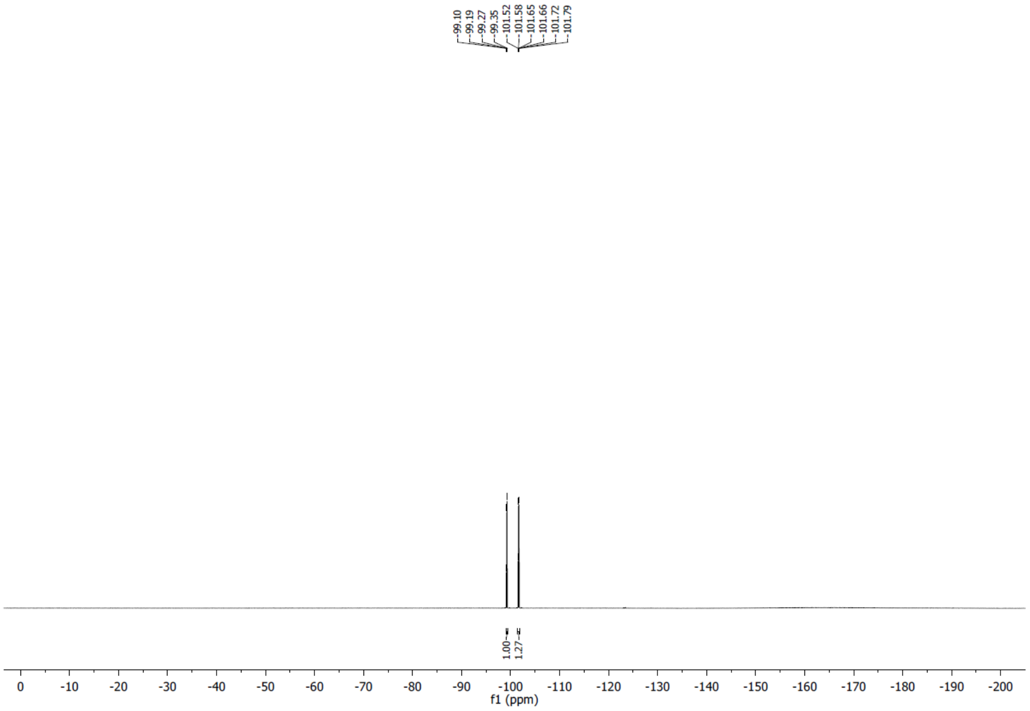

HRMS-spectrum

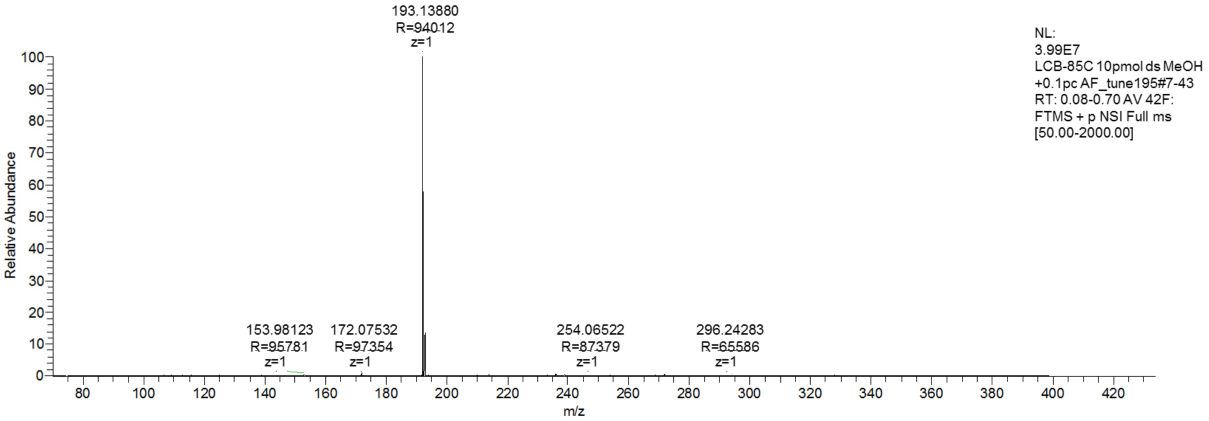

(*E*/*Z*)-1-(2-fluorohex-1-en-1-yl)-4-methoxybenzene 3da

^{1}H NMR Spectrum ($CDCl_3$, 300 MHz)

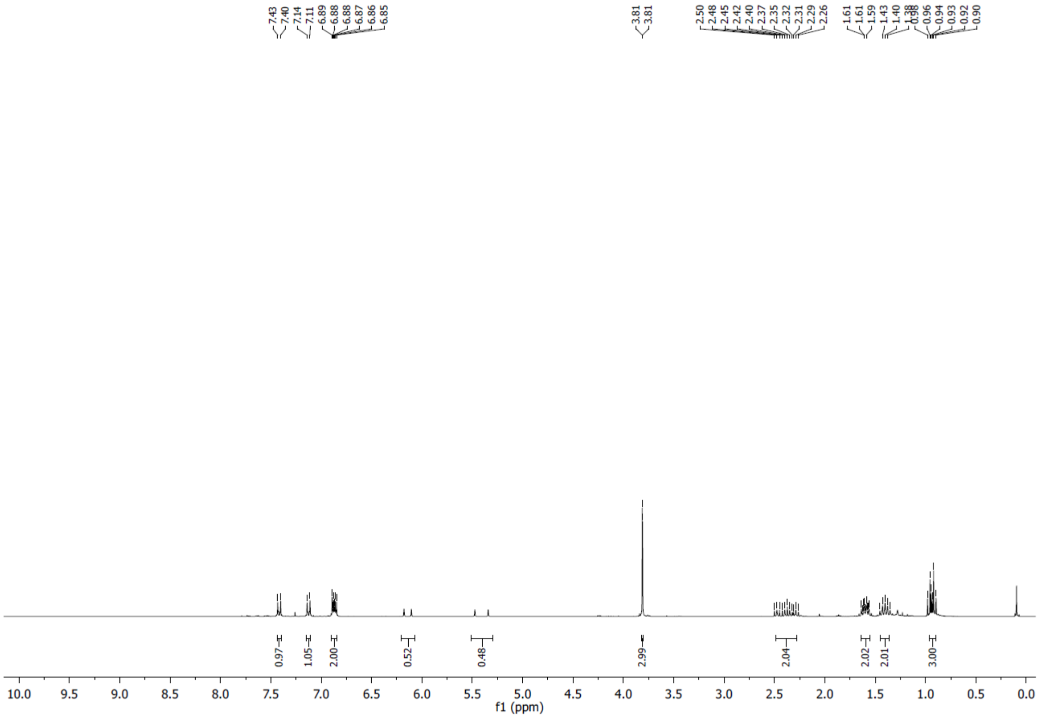

^{13}C ($CDCl_3$, 75 MHz)

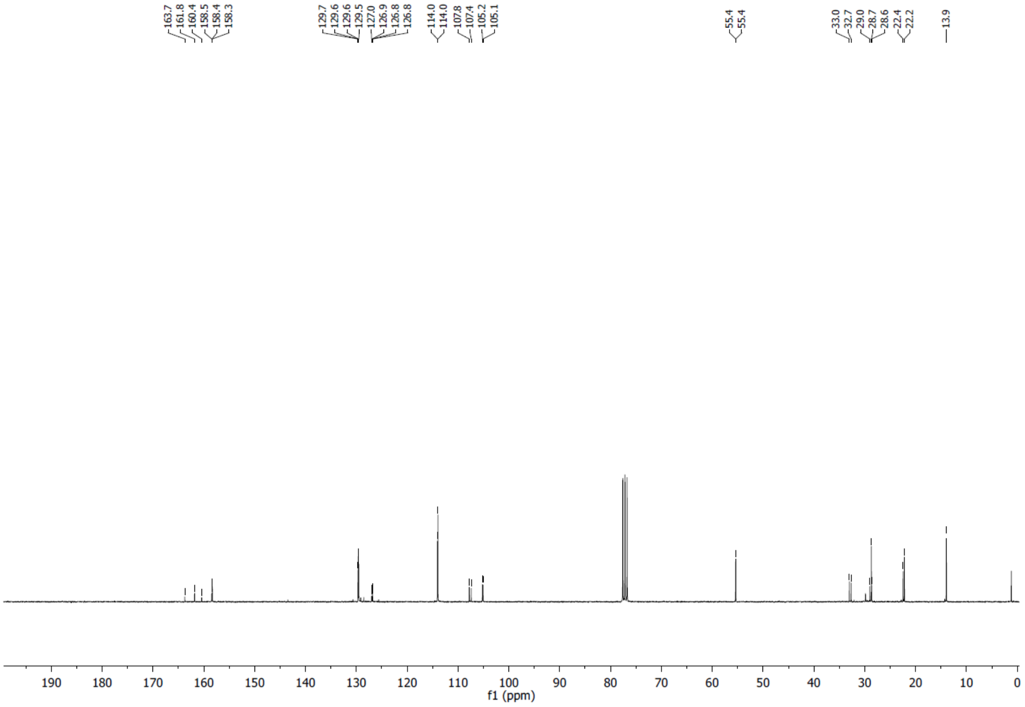

^{19}F NMR (282.5 MHz, CDCl$_3$)

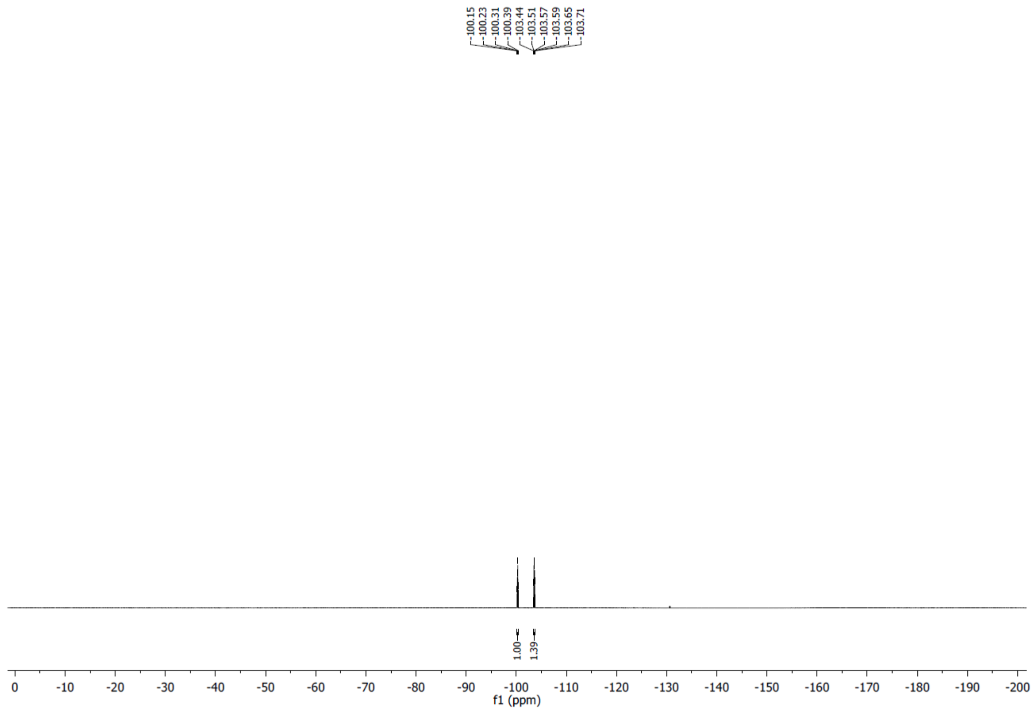

HRMS-spectrum

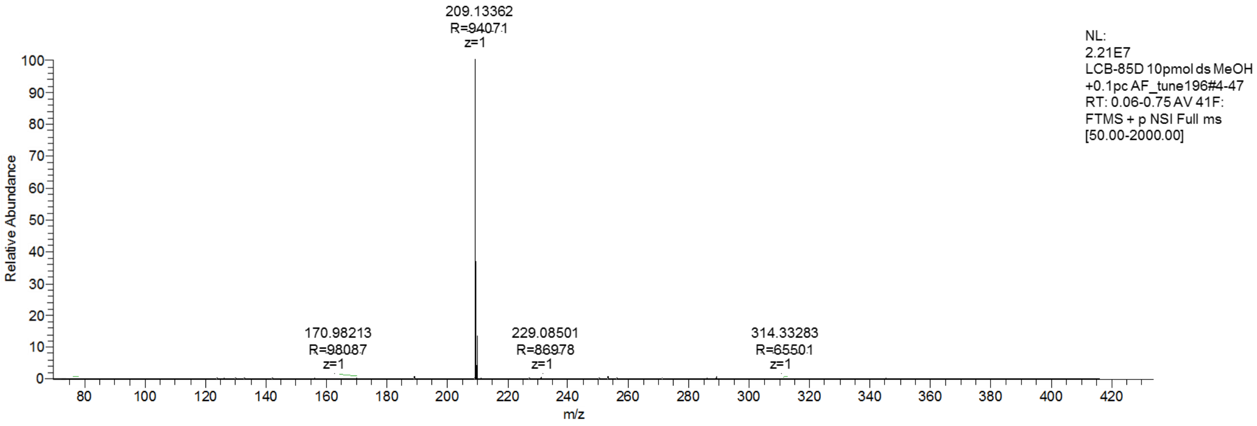

<u>(*E*/*Z*)-methyl 4-(2-fluorohex-1-en-1-yl)benzoate 3ea</u>

^{1}H NMR Spectrum (CDCl$_3$, 300 MHz)

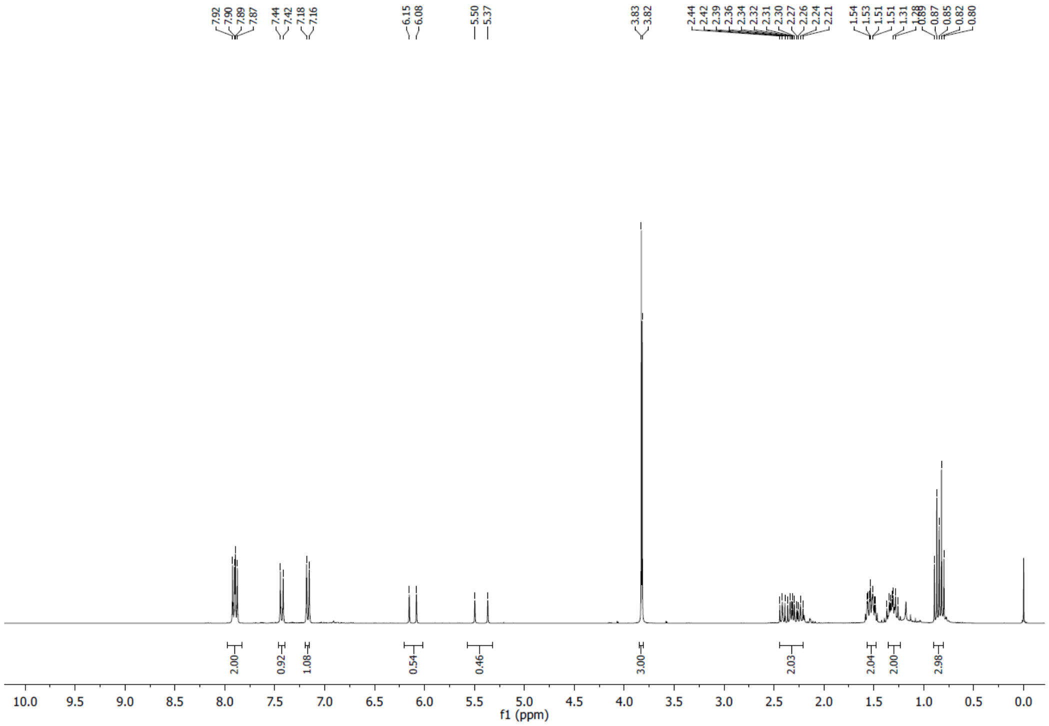

^{13}C (CDCl$_3$, 75 MHz)

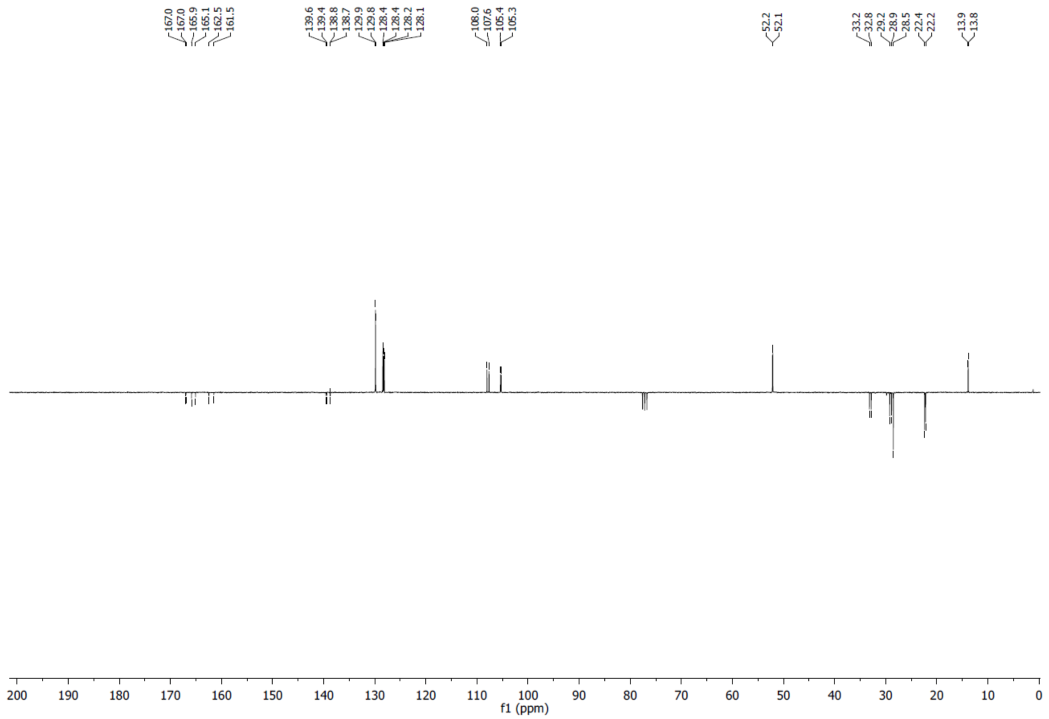

^{19}F NMR (282.5 MHz, $CDCl_3$)

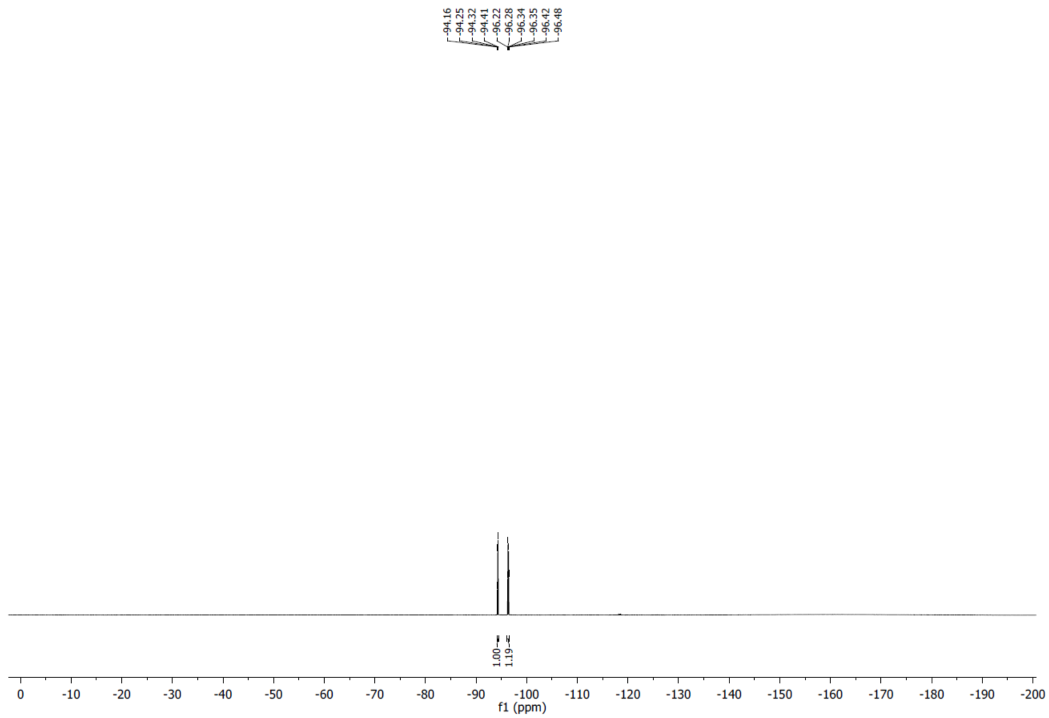

HRMS-spectrum

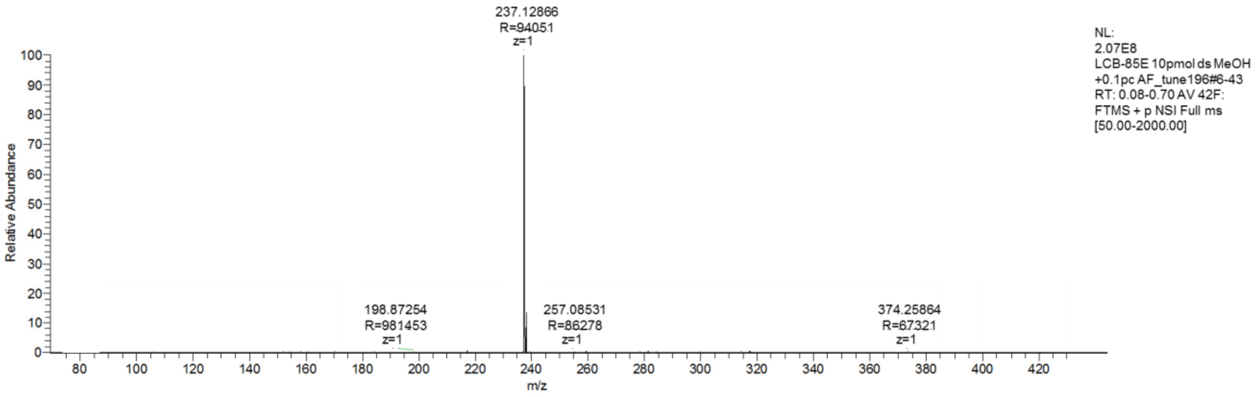
237.12866
R=94051
z=1
NL:
2.07E8
LCB-85E 10pmol ds MeOH
+0.1pc AF_tune196#6-43
RT: 0.08-0.70 AV 42F:
FTMS + p NSI Full ms
[50.00-2000.00]
198.87254
R=981453
z=1
257.08531
R=86278
z=1
374.25864
R=67321
z=1
Relative Abundance
m/z

(*E*/*Z*)-1-(2-fluorohex-1-en-1-yl)-4-(trifluoromethyl)benzene 3fa

^{1}H NMR Spectrum ($CDCl_3$, 300 MHz)

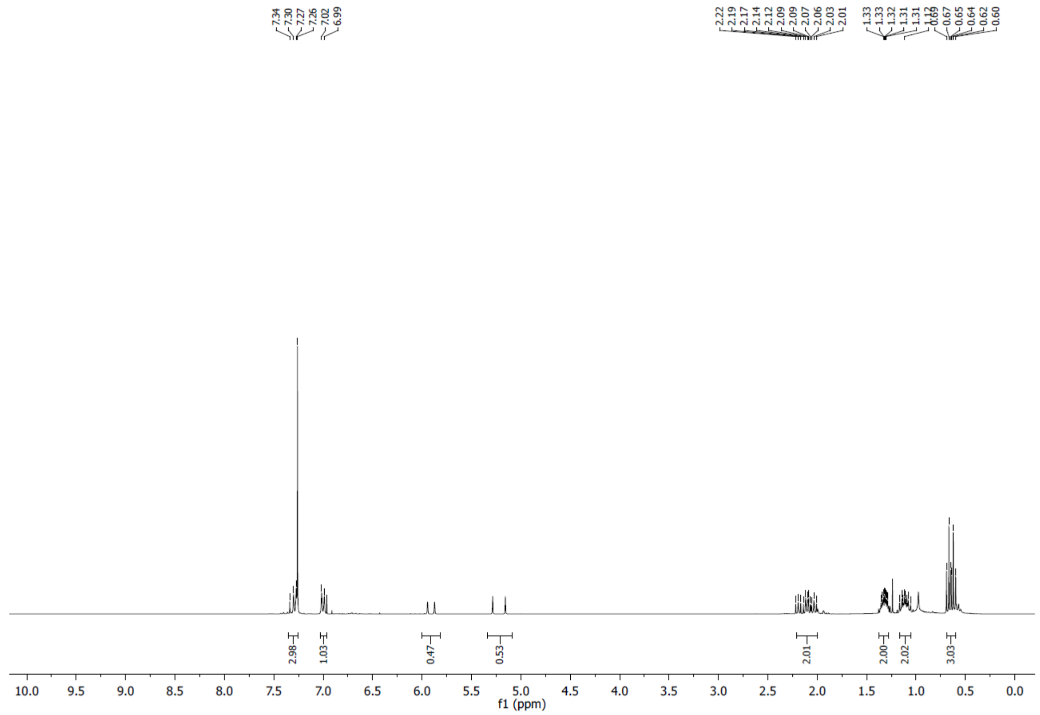

^{13}C ($CDCl_3$, 75 MHz)

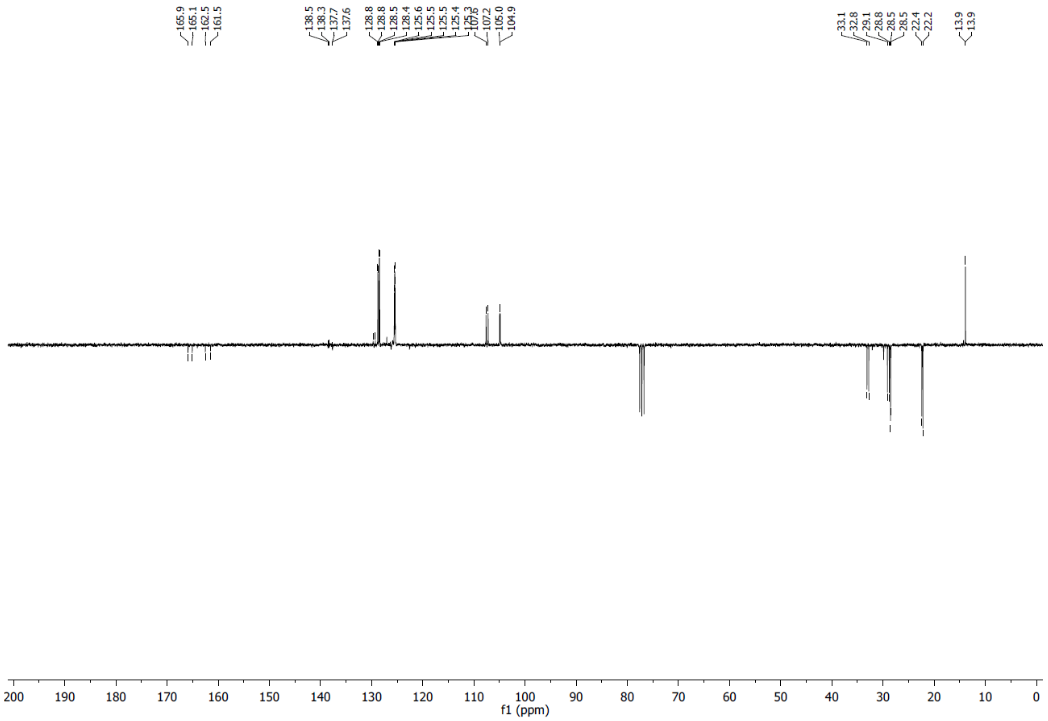

^{19}F NMR (282.5 MHz, $CDCl_3$)

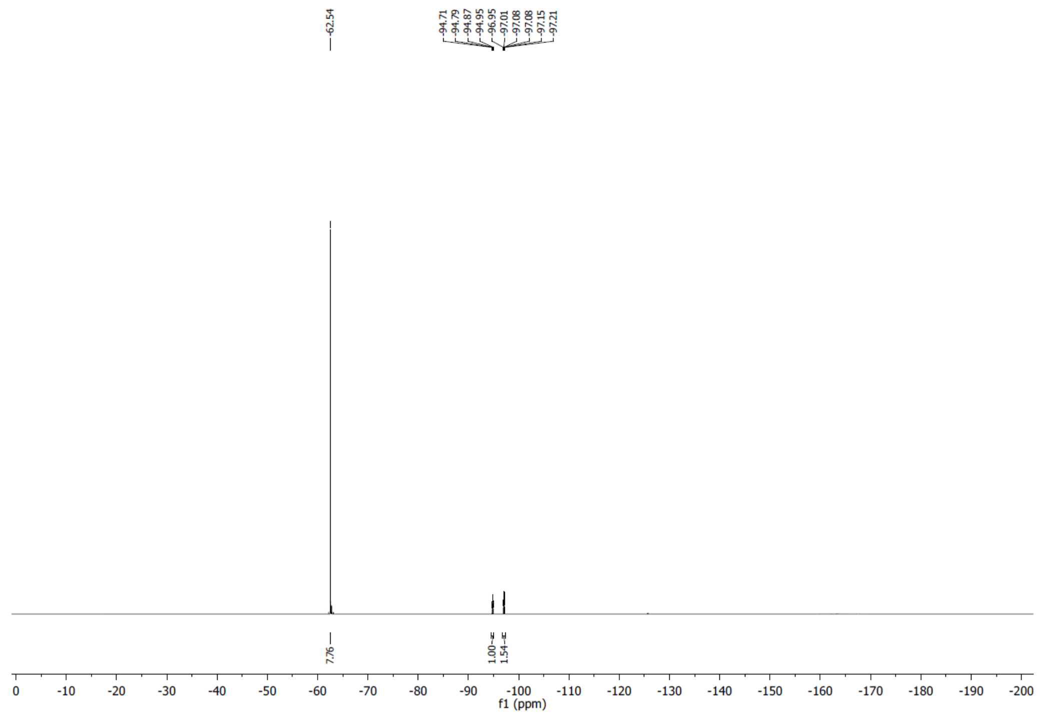

HRMS-spectrum

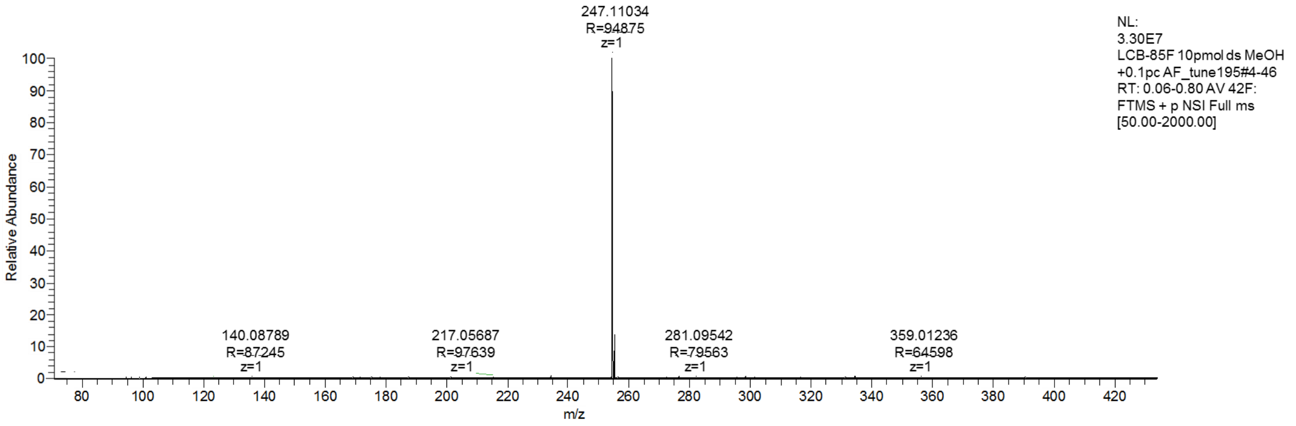

<u>(*E*/*Z*)-1-chloro-4-(2-fluorohex-1-en-1-yl)benzene 3ga</u>

^1H NMR Spectrum ($CDCl_3$, 300 MHz)

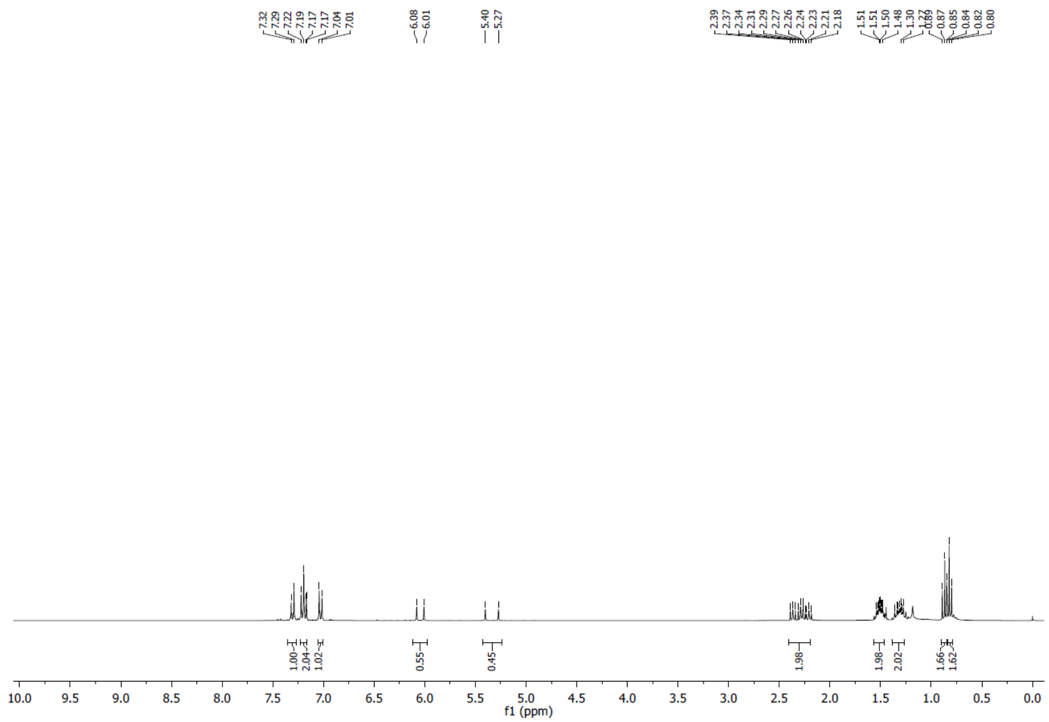

^{13}C ($CDCl_3$, 75 MHz)

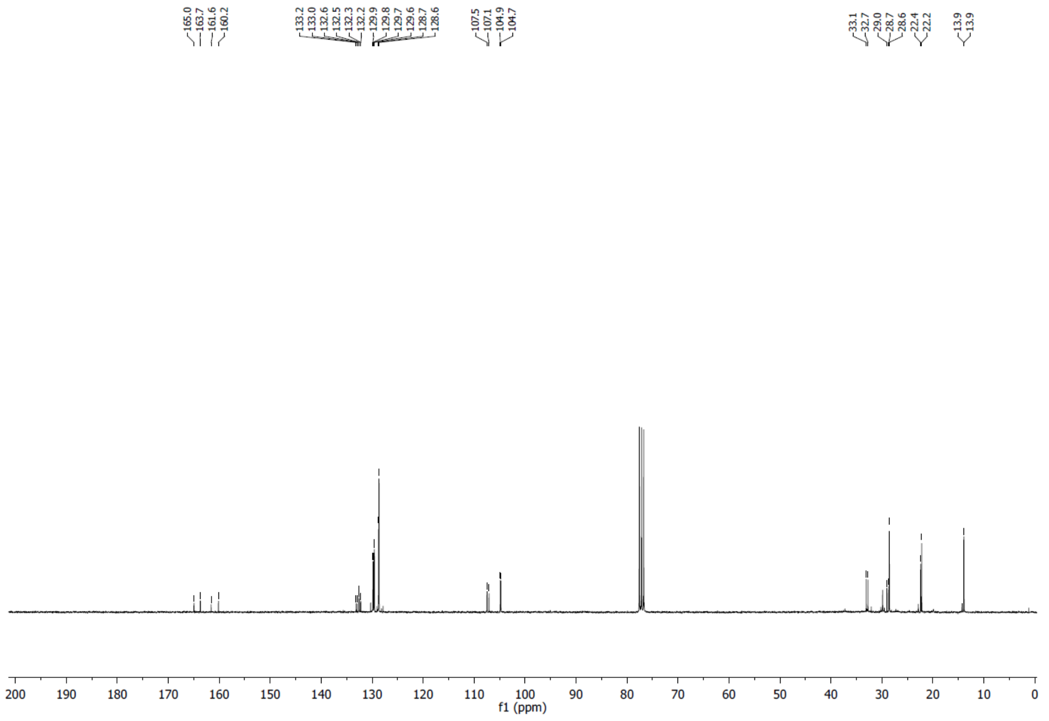

^{19}F NMR (282.5 MHz, $CDCl_3$)

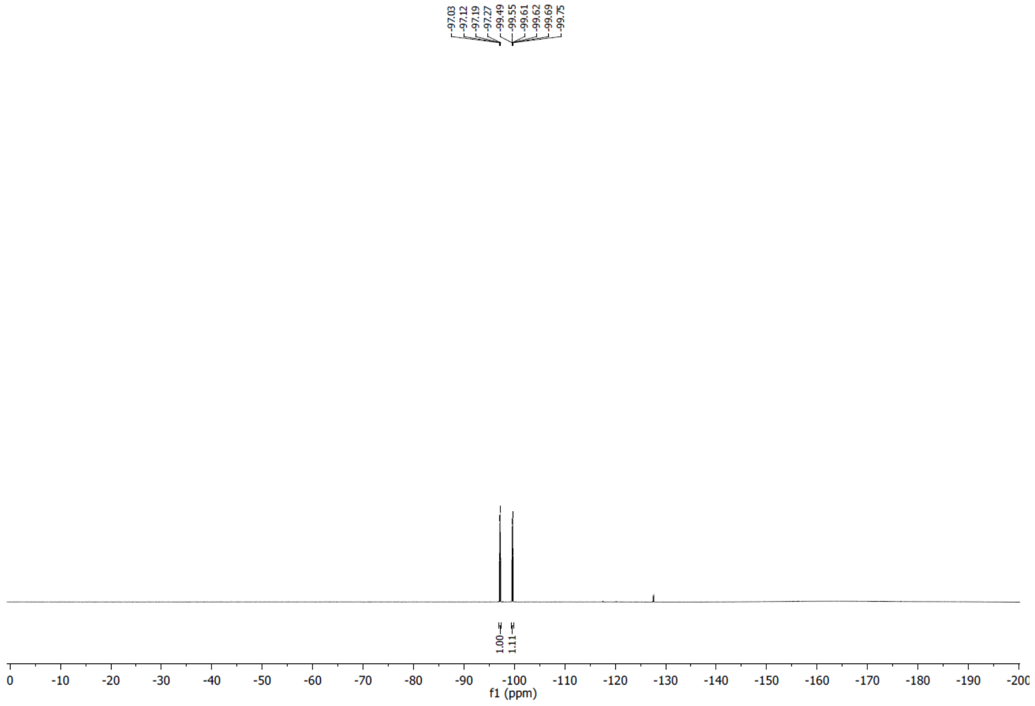

HRMS-spectrum

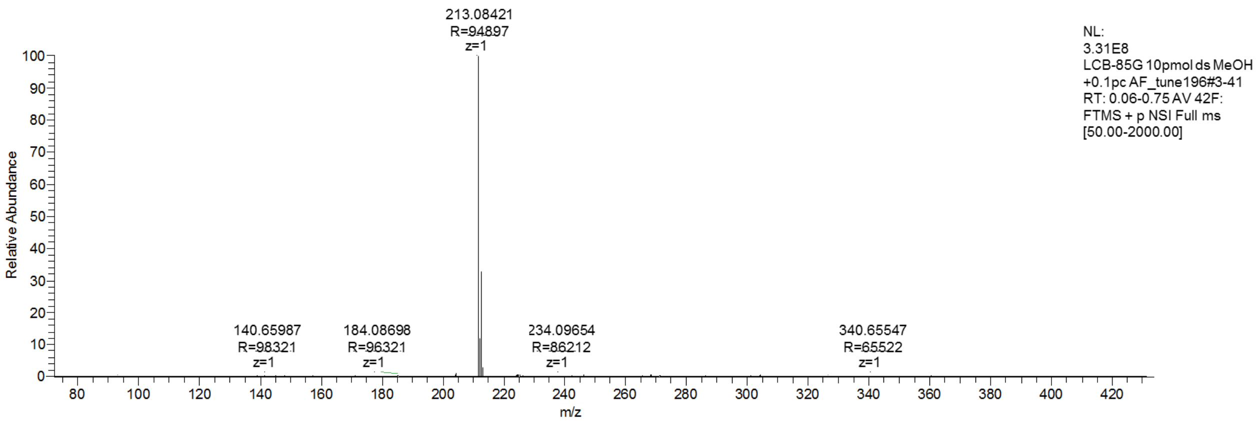

(*E*/*Z*)-1-(2-fluorohex-1-en-1-yl)-3-nitrobenzene 3ha

^{1}H NMR Spectrum (CDCl$_3$, 300 MHz)

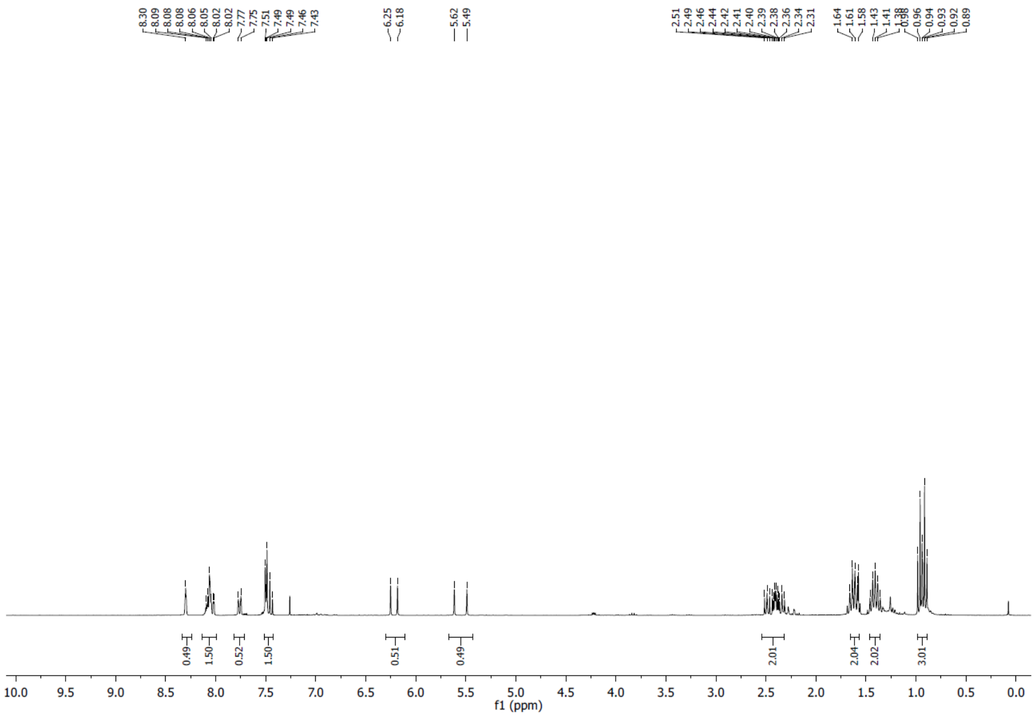

^{13}C (CDCl$_3$, 75 MHz)

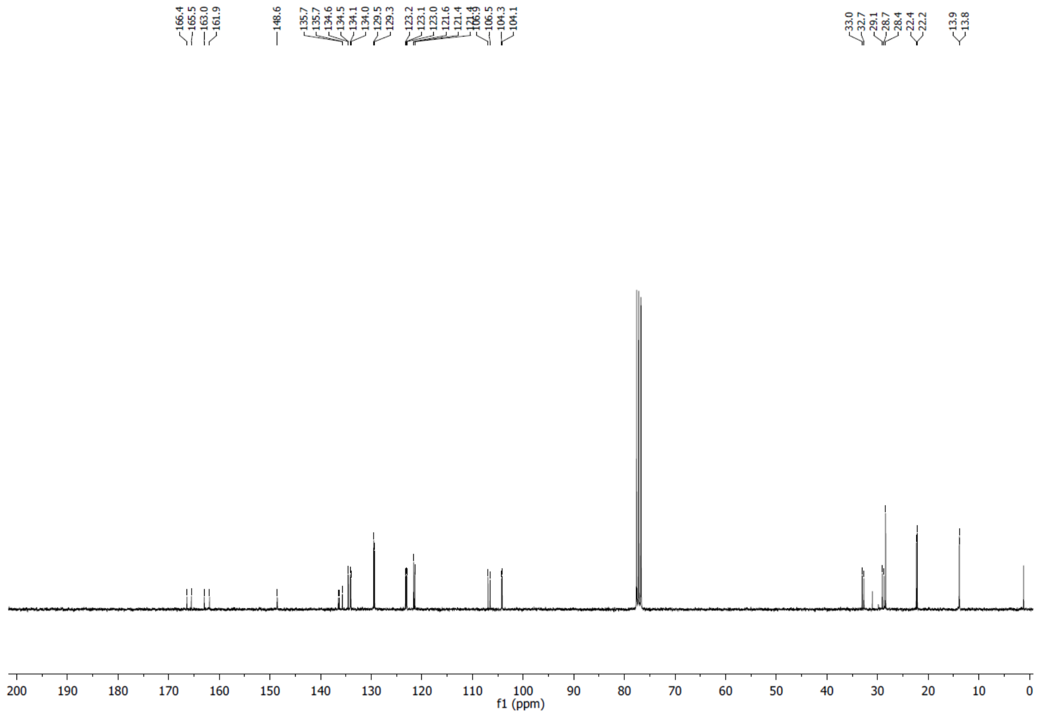

^{19}F NMR (282.5 MHz, CDCl$_3$)

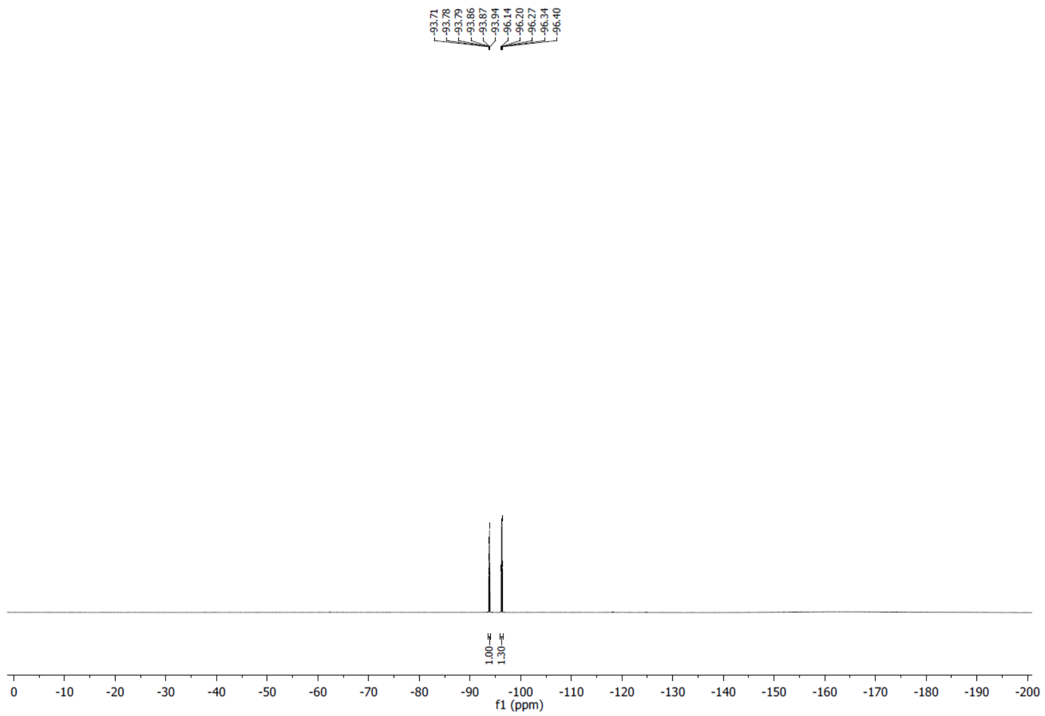

HRMS-spectrum

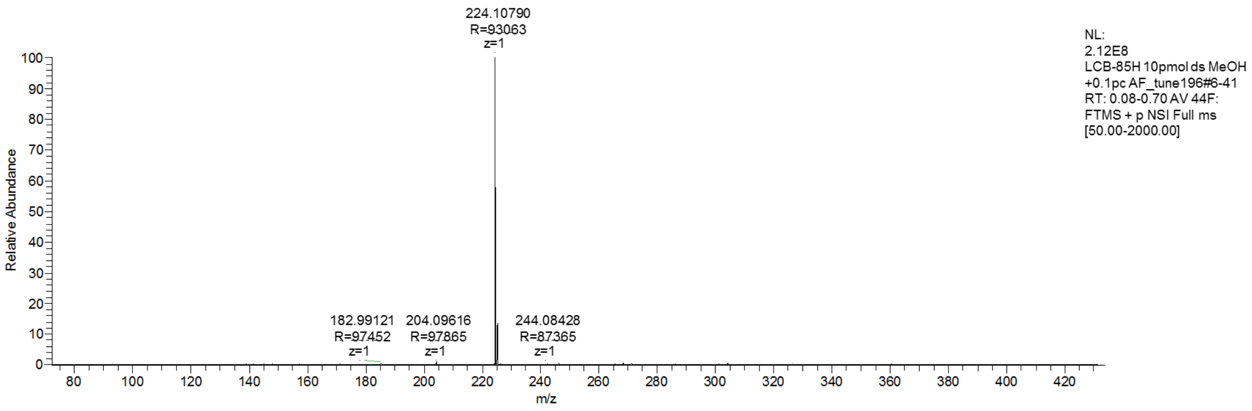

(*E*/*Z*)-1-(2-fluorohex-1-en-1-yl)-2-methoxybenzene 3ia

^{1}H NMR Spectrum ($CDCl_3$, 300 MHz)

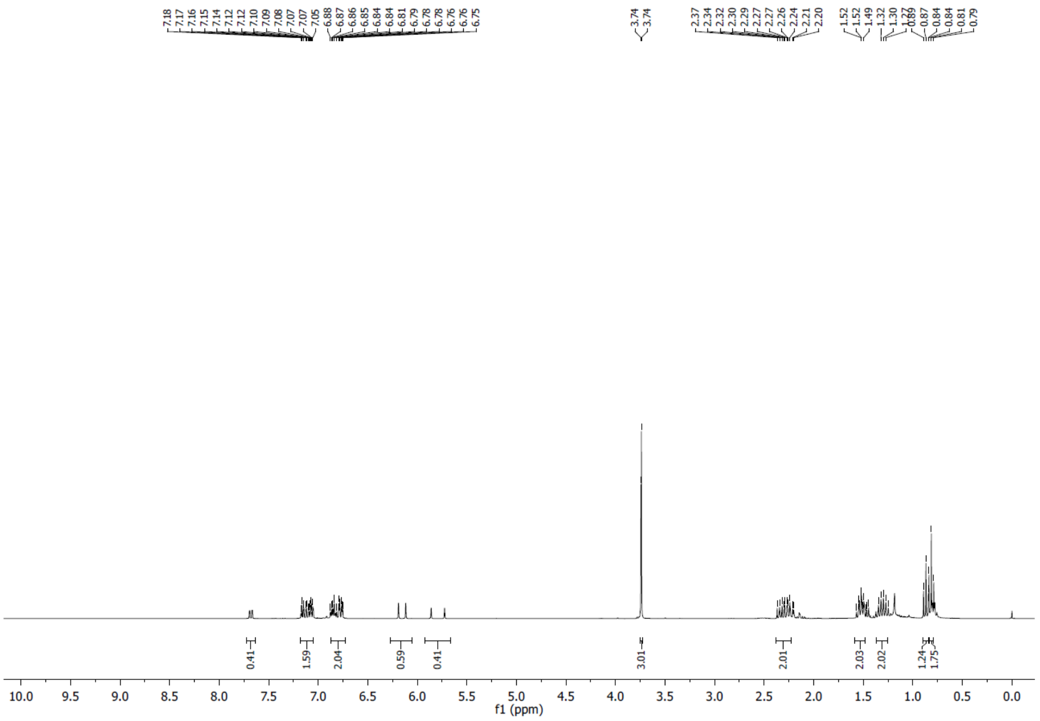

^{13}C ($CDCl_3$, 75 MHz)

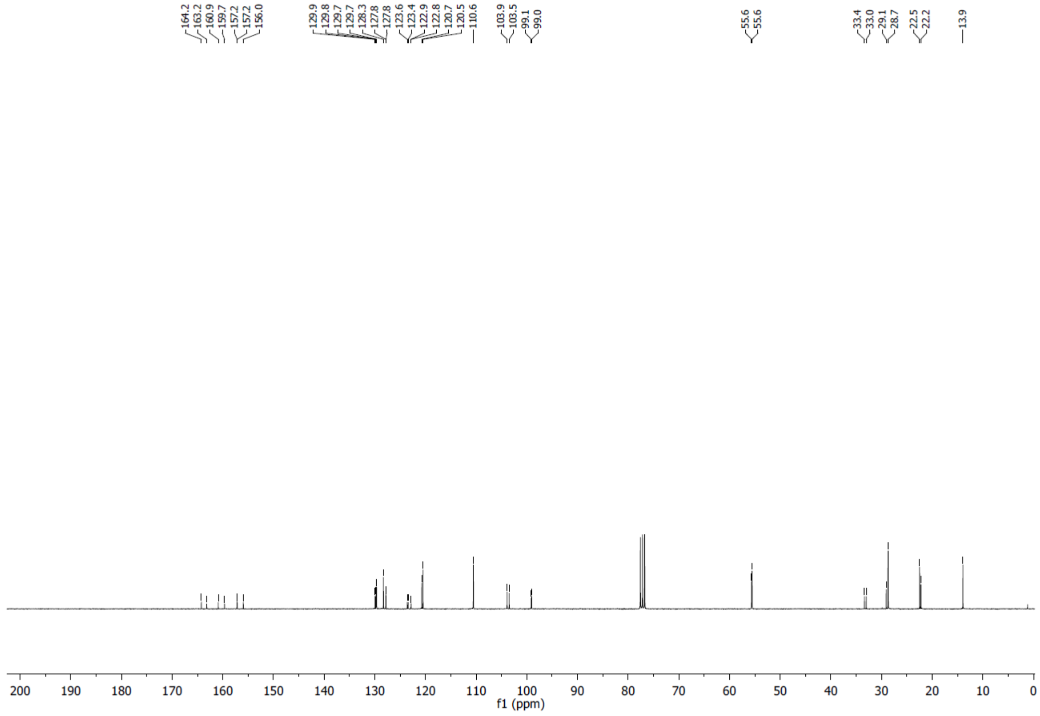

^{19}F NMR (282.5 MHz, CDCl$_3$)

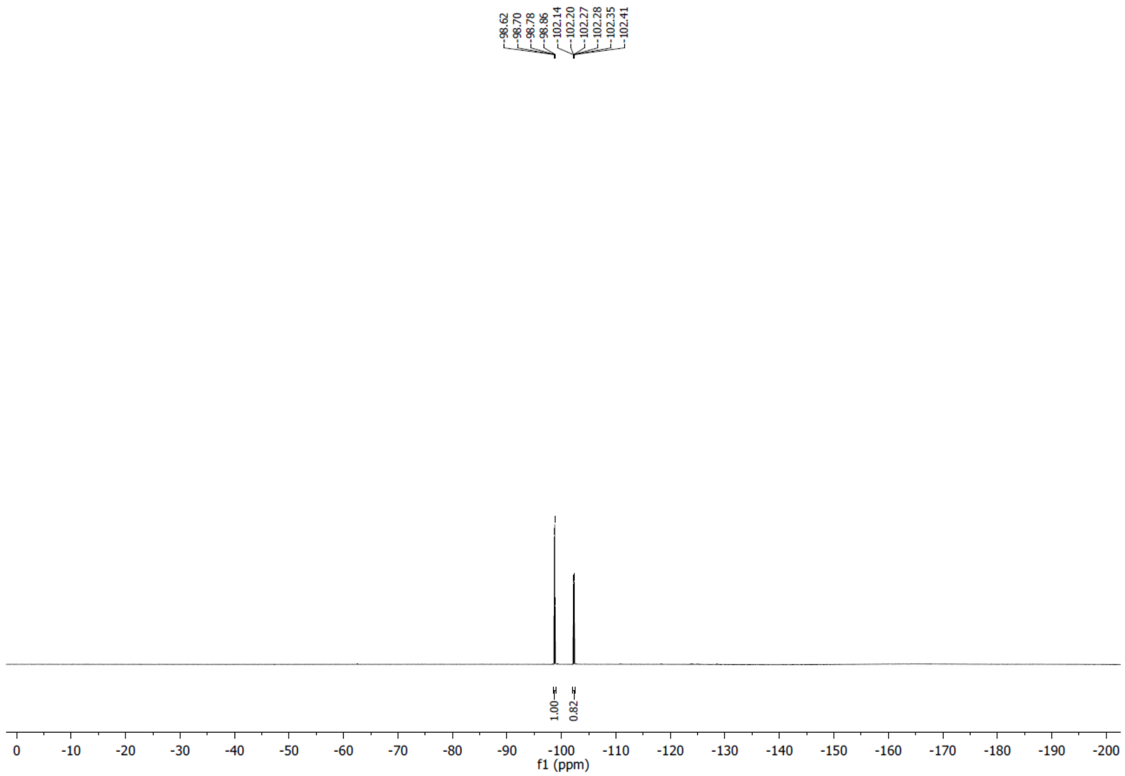

HRMS-spectrum

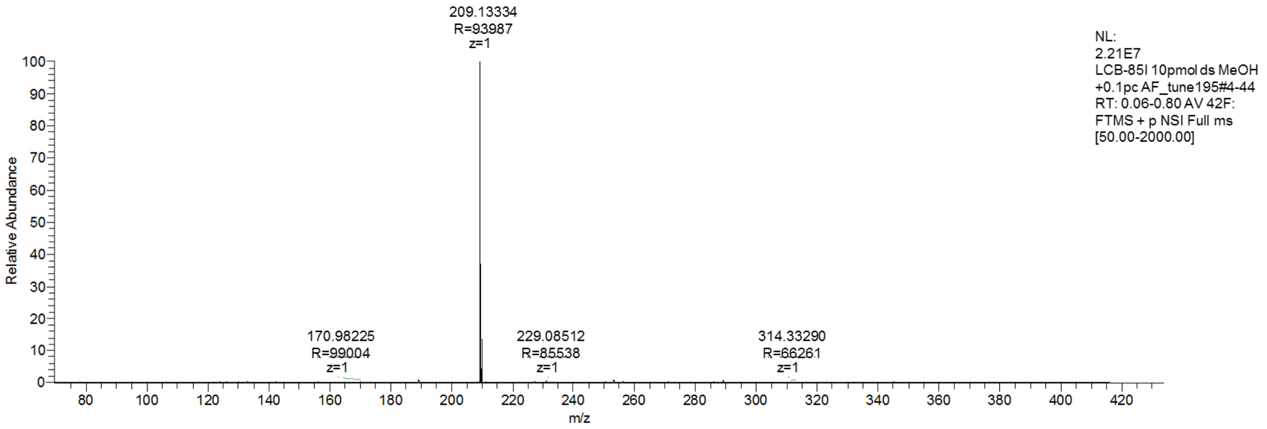

(2-bromo-2-fluoroethene-1,1-diyl)dibenzene 3ja

^{1}H NMR Spectrum (CDCl$_3$, 300 MHz)

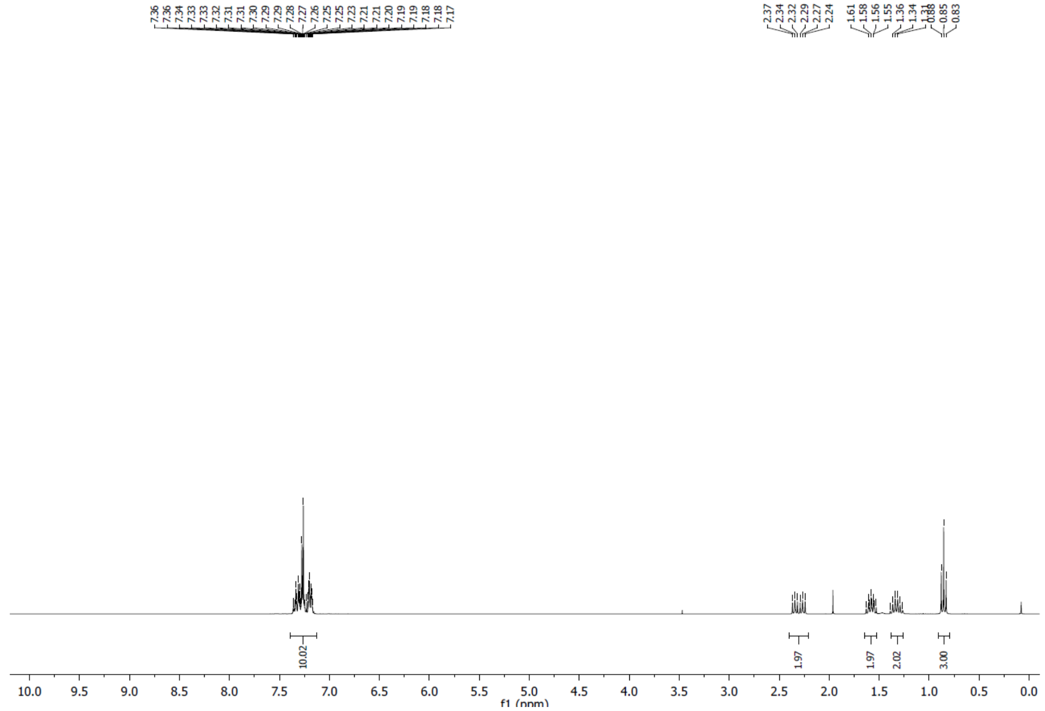

^{13}C (CDCl$_3$, 75 MHz)

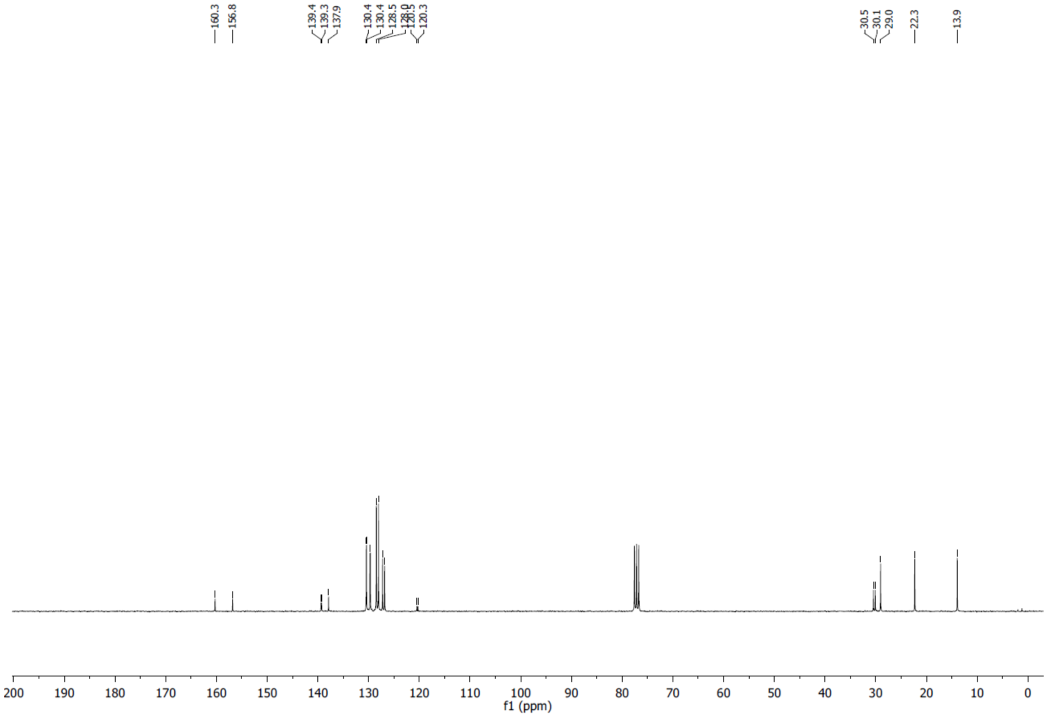

^{19}F NMR (282.5 MHz, CDCl$_3$)

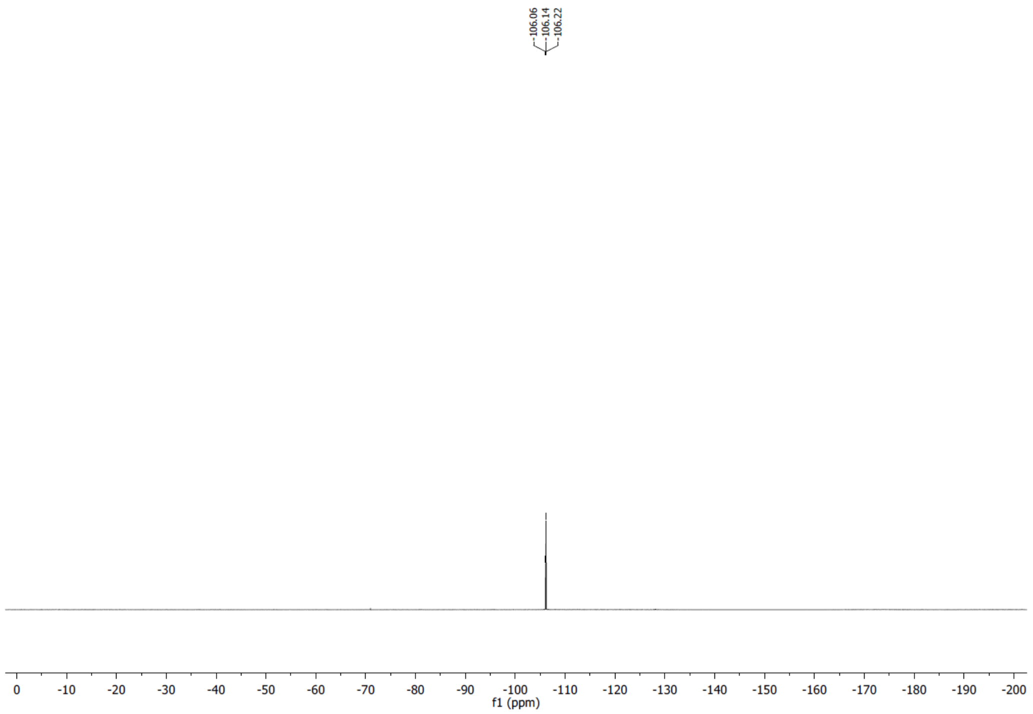

HRMS-spectrum

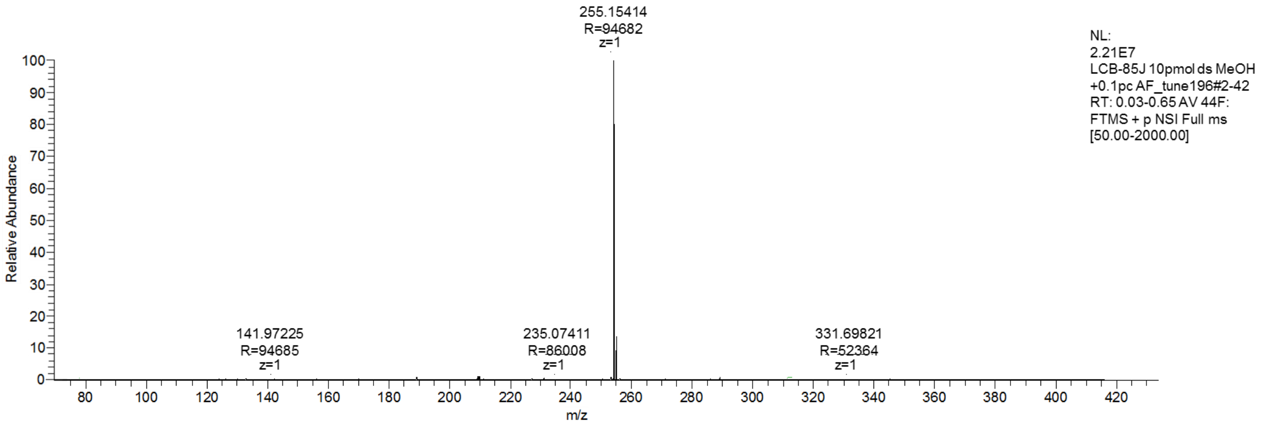

(*E*/*Z*)-1-(3-fluorohept-2-en-2-yl)-4-methoxybenzene 3ka

^{1}H NMR Spectrum ($CDCl_3$, 300 MHz)

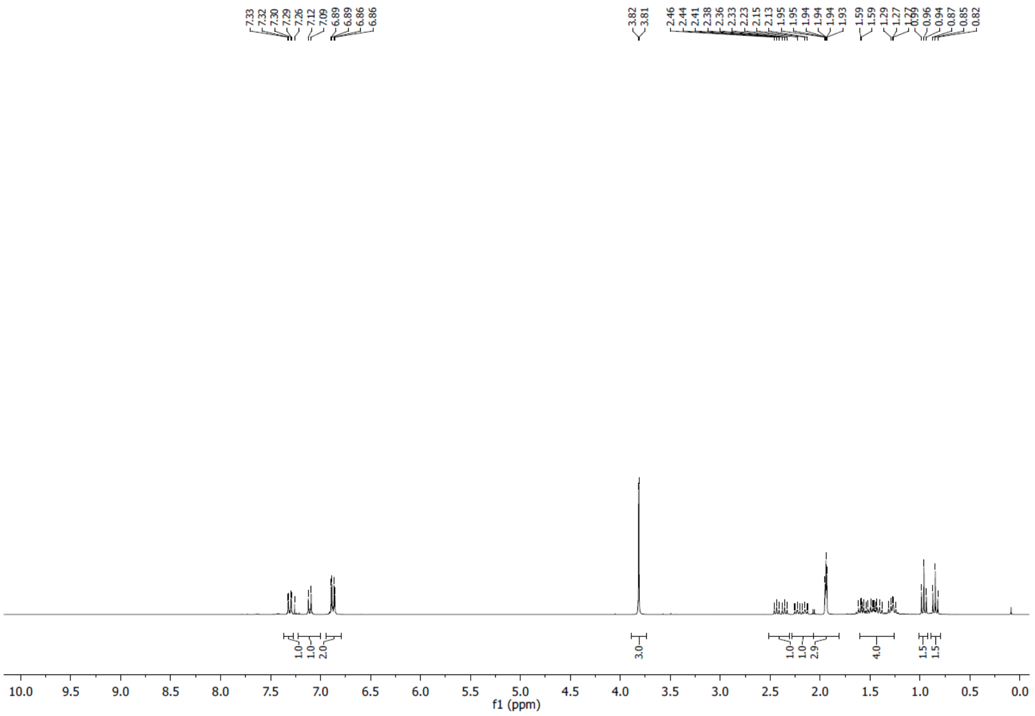

^{13}C ($CDCl_3$, 75 MHz)

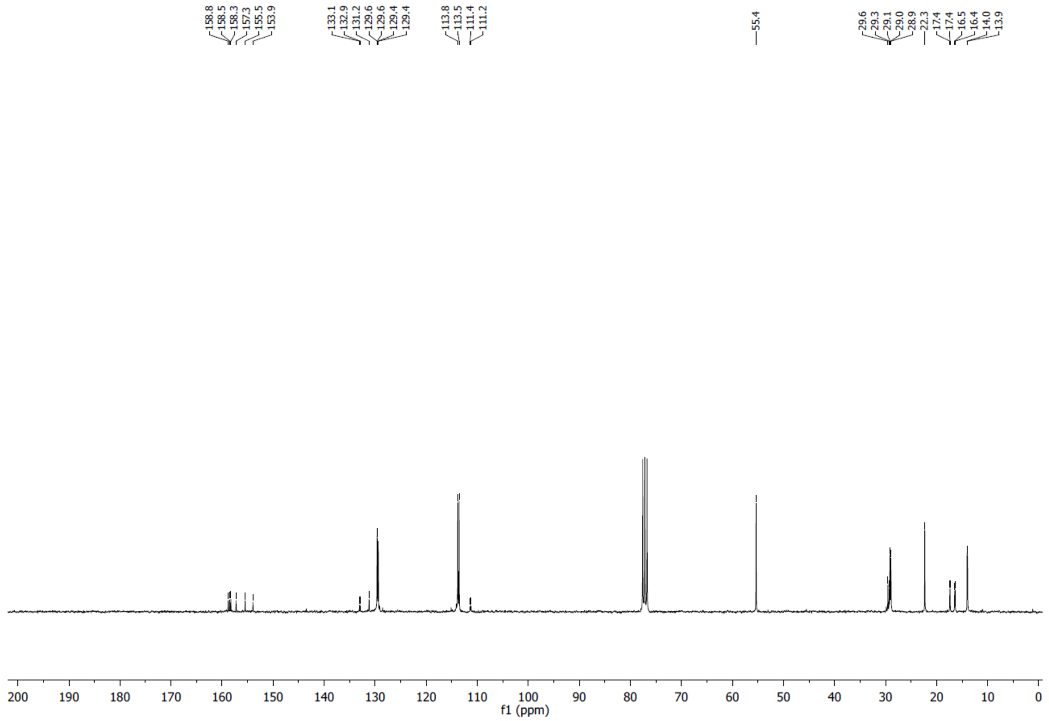

^{19}F NMR (282.5 MHz, $CDCl_3$)

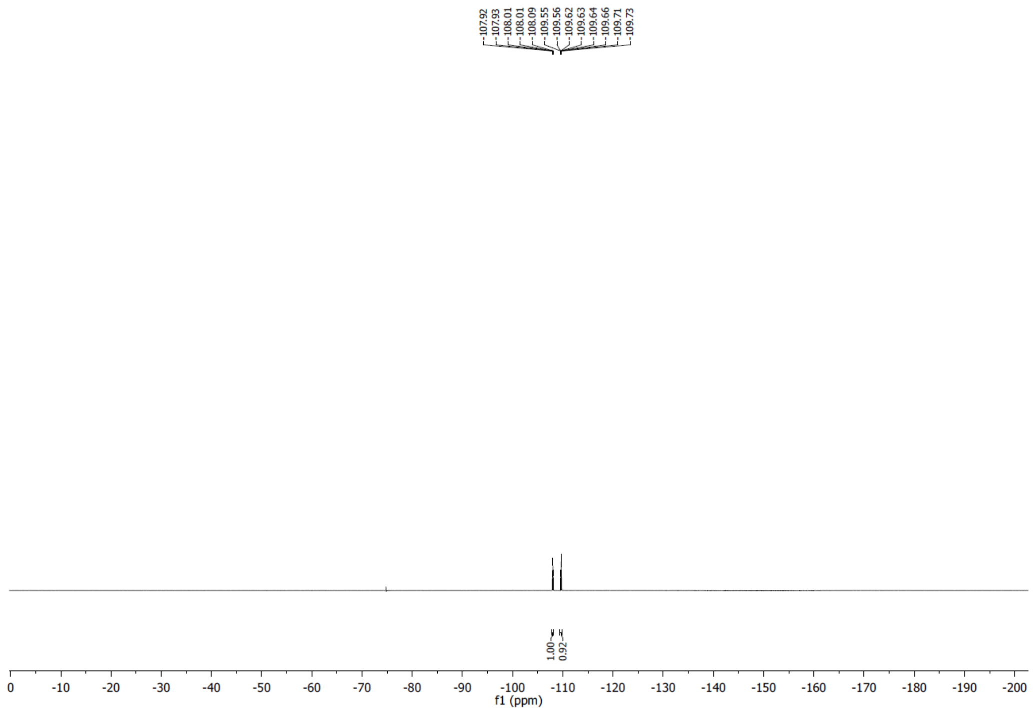

HRMS-spectrum

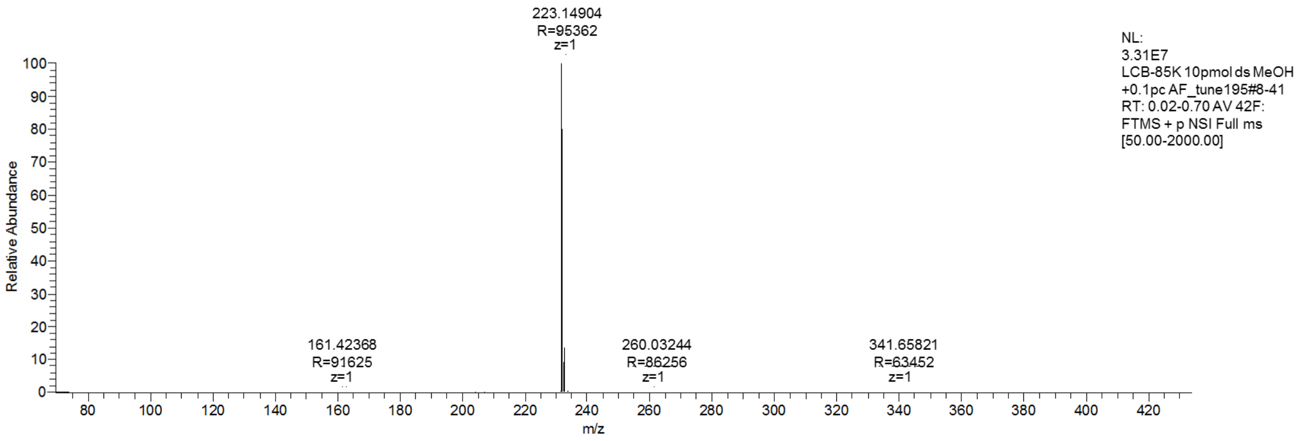
223.14904
R=95362
z=1
161.42368
R=91625
z=1
260.03244
R=86256
z=1
341.65821
R=63452
z=1
NL:
3.31E7
LCB-85K 10pmol ds MeOH
+0.1pc AF_tune195#8-41
RT: 0.02-0.70 AV 42F:
FTMS + p NSI Full ms
[50.00-2000.00]
Relative Abundance
m/z

1-(2-fluoropent-1-en-1-yl)-4-nitrobenzene 3ac

^{1}H NMR Spectrum ($CDCl_3$, 300 MHz)

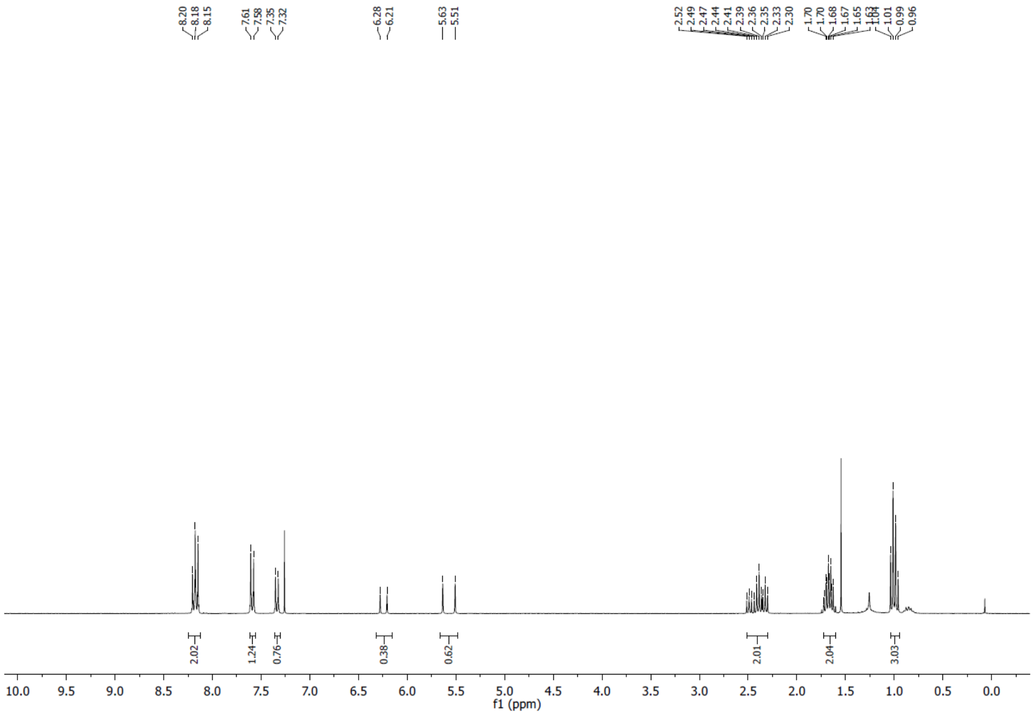

^{13}C ($CDCl_3$, 75 MHz)

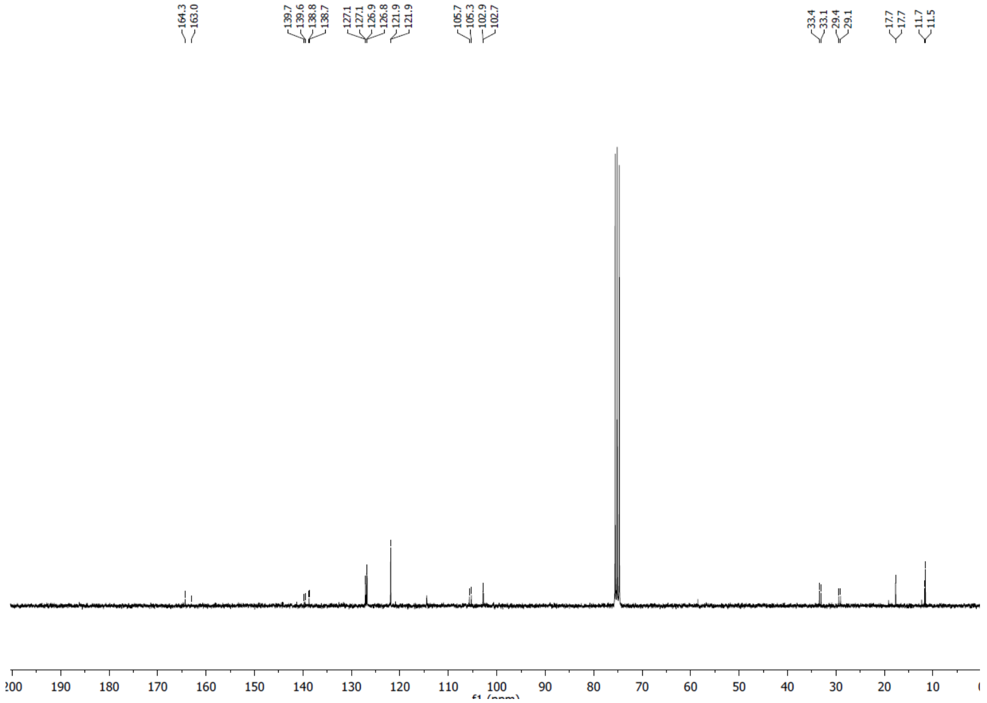

^{19}F NMR (282.5 MHz, $CDCl_3$)

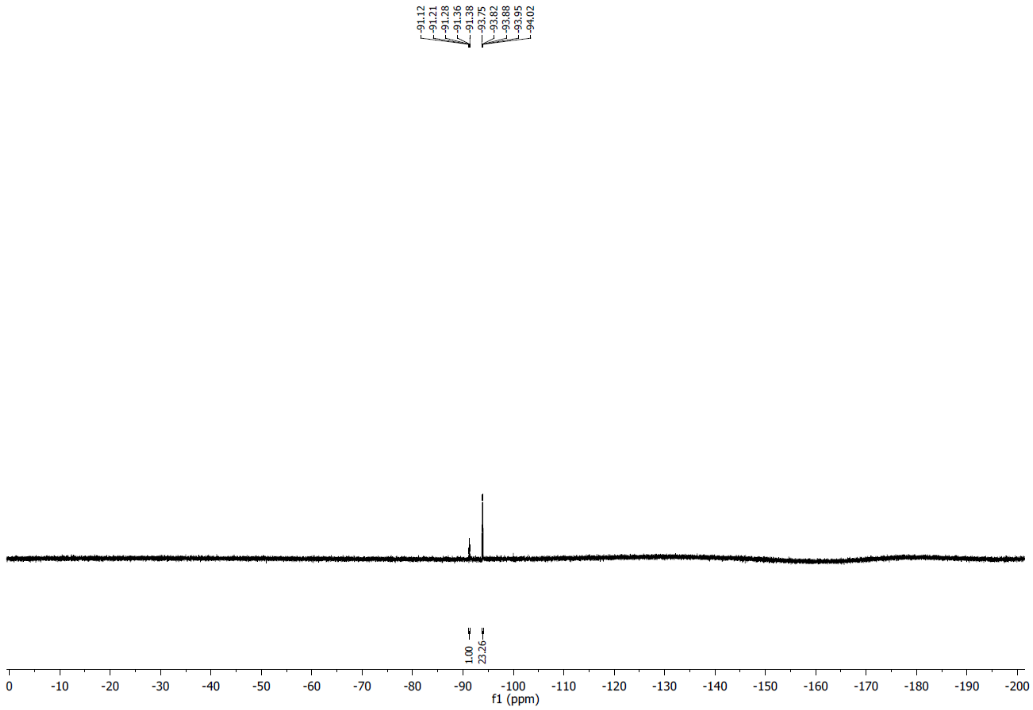

HRMS-spectrum

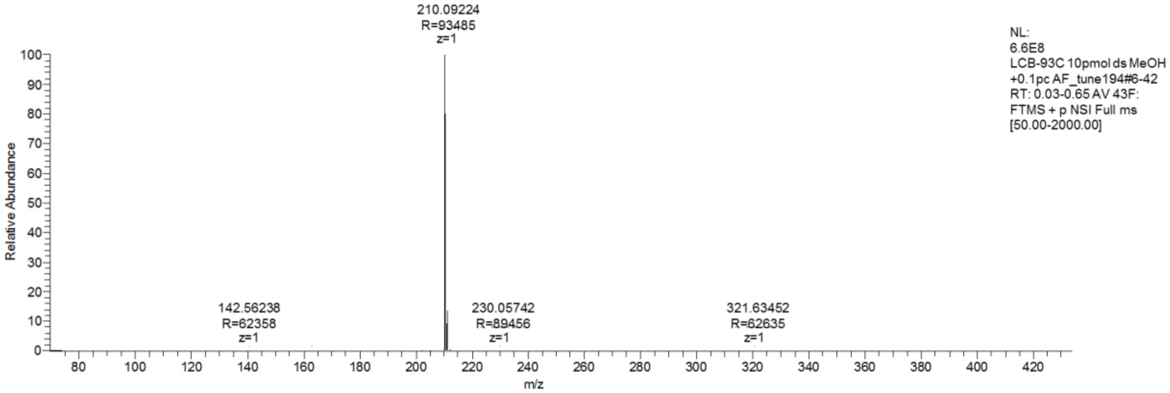

__1-(2-fluoro-4-phenylbut-1-en-1-yl)-4-nitrobenzene 3ad__

^{1}H NMR Spectrum ($CDCl_3$, 300 MHz)

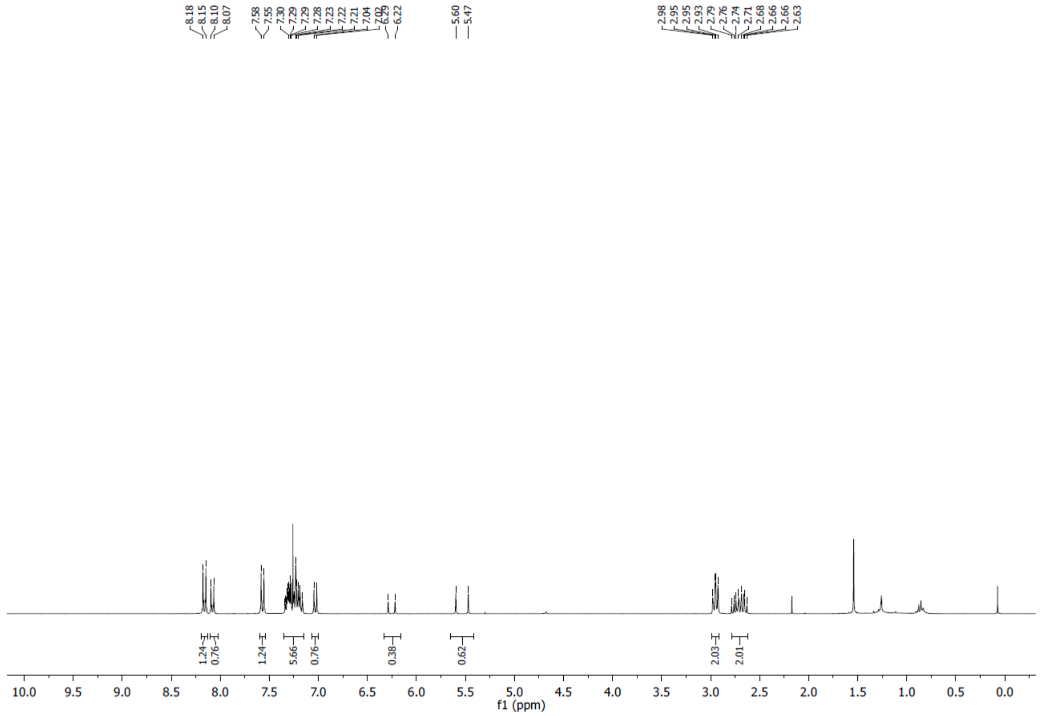

^{13}C ($CDCl_3$, 75 MHz)

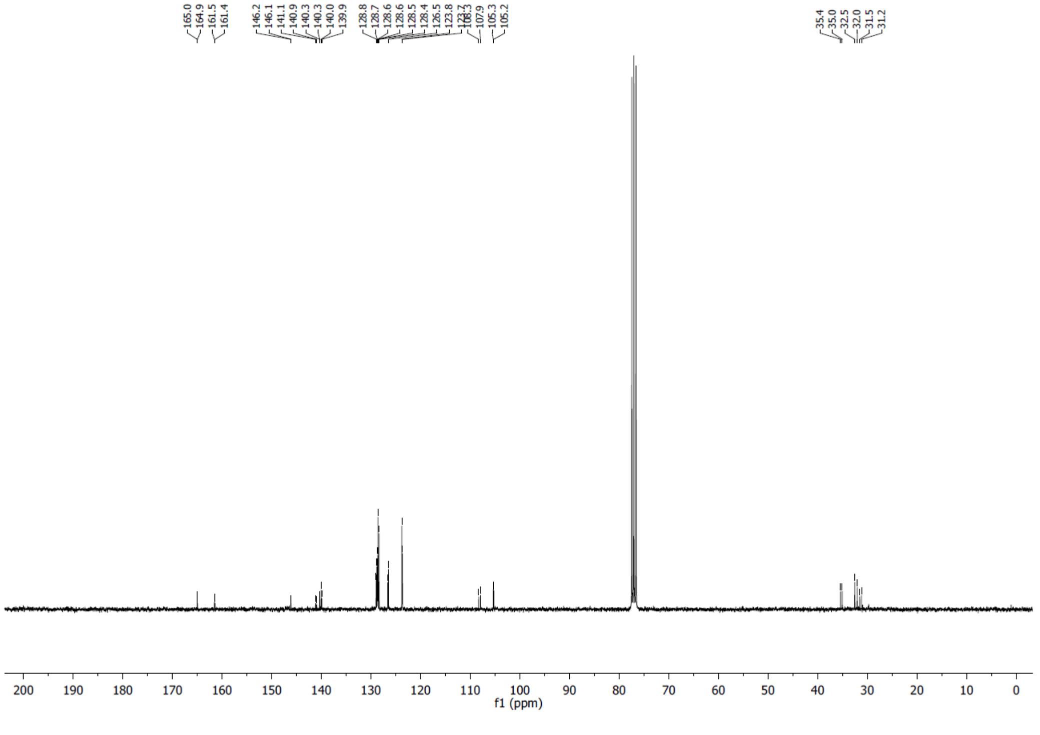

^{19}F NMR (282.5 MHz, $CDCl_3$)

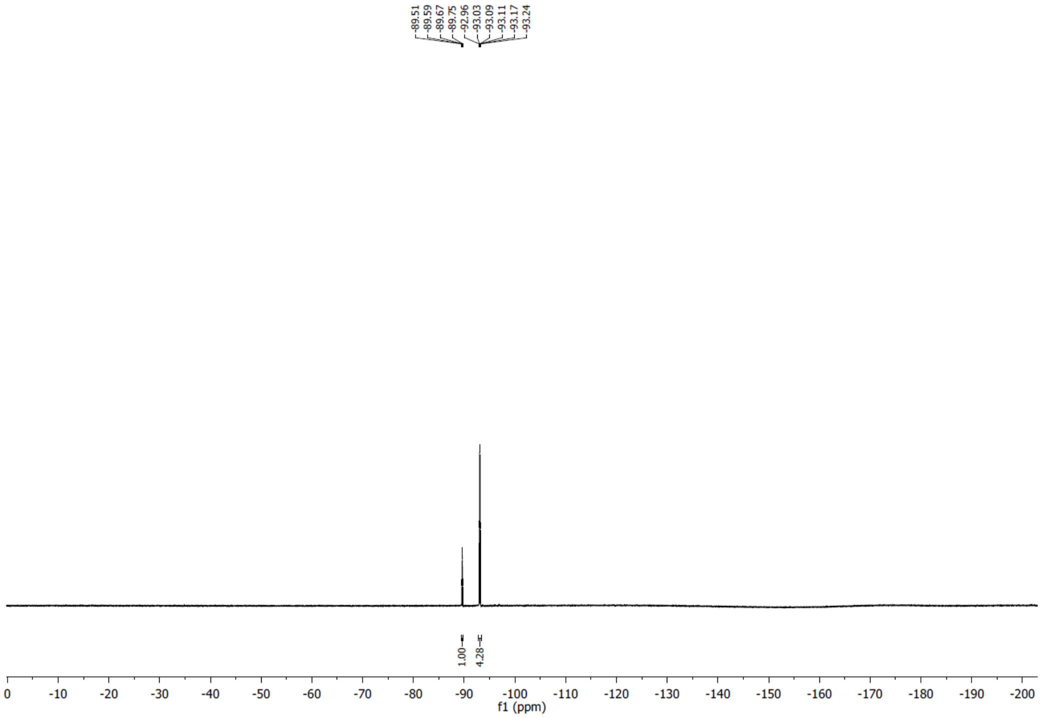

HRMS-spectrum

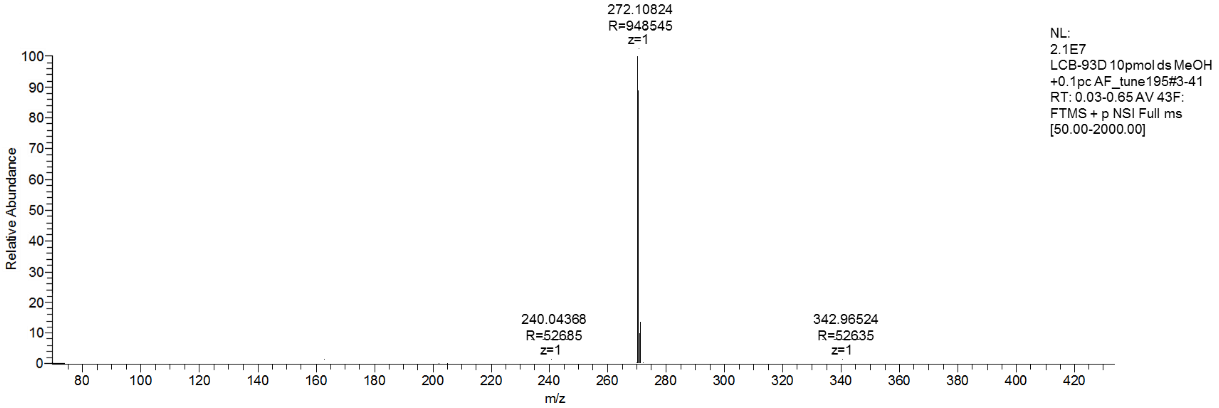

1-(2-fluoro-4-methylpent-1-en-1-yl)-4-nitrobenzene 3ae

^{1}H NMR Spectrum ($CDCl_3$, 300 MHz)

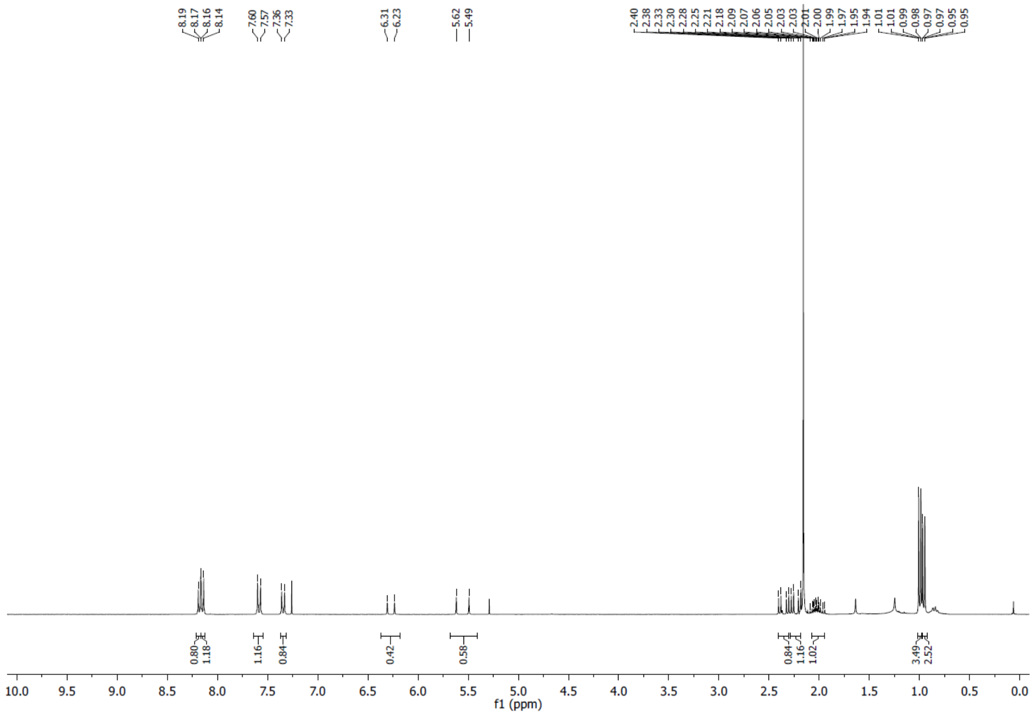

^{13}C ($CDCl_3$, 75 MHz)

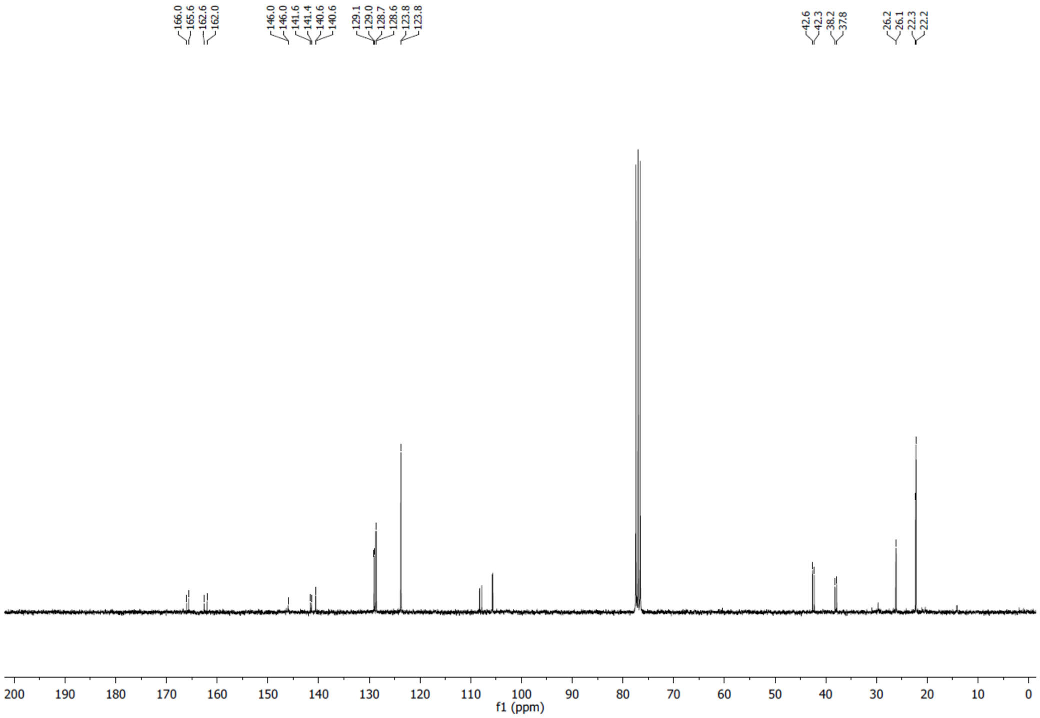

^{19}F NMR (282.5 MHz, $CDCl_3$)

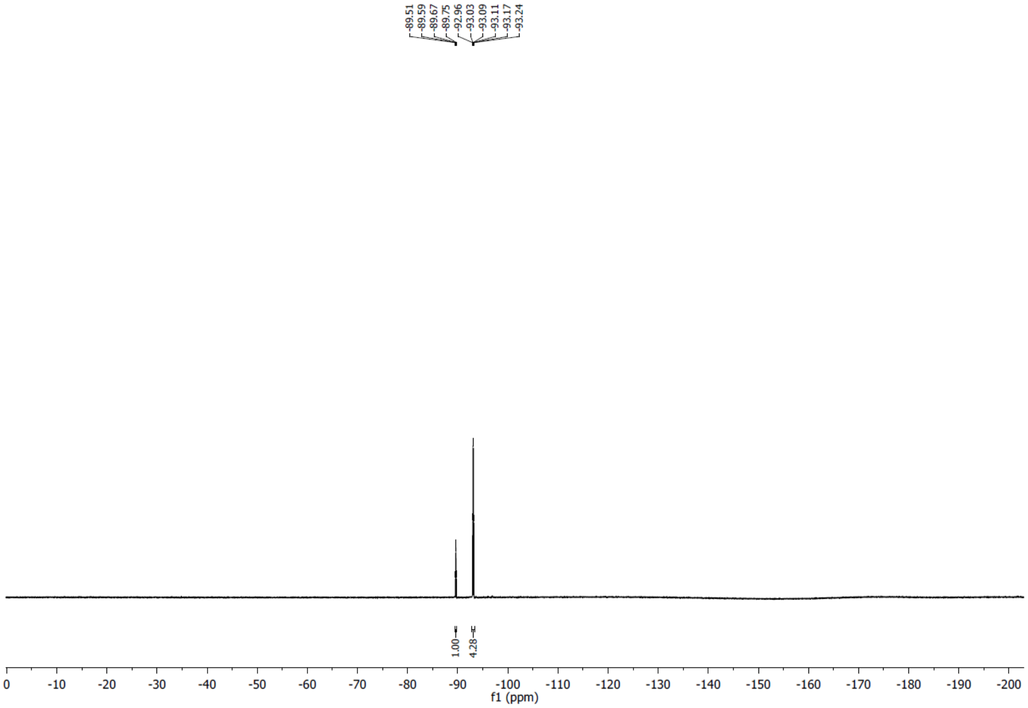

HRMS-spectrum

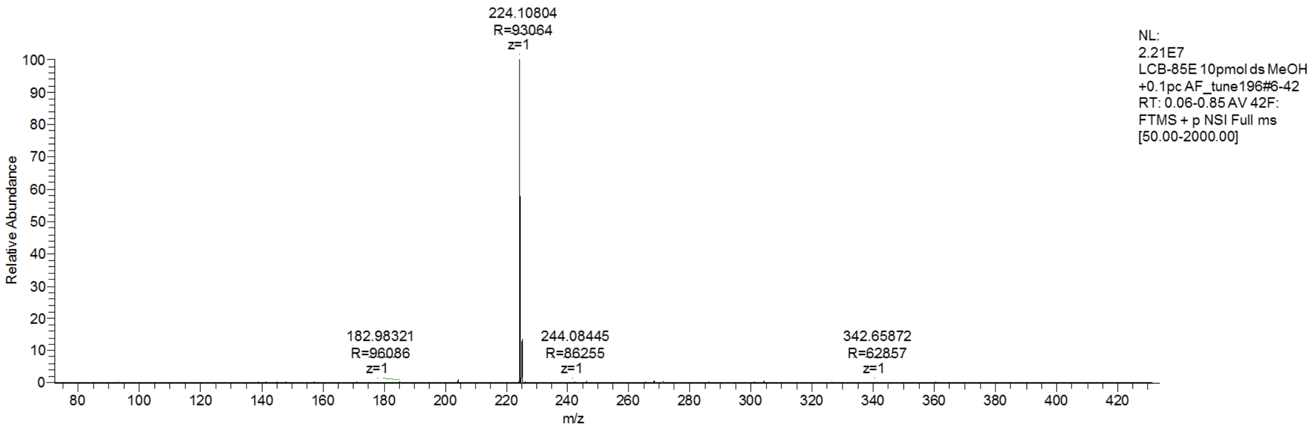
224.10804
R=93064
z=1
182.98321
R=96086
z=1
244.08445
R=86255
z=1
342.65872
R=62857
z=1
NL:
2.21E7
LCB-85E 10pmol ds MeOH
+0.1pc AF_tune196#6-42
RT: 0.06-0.85 AV 42F:
FTMS + p NSI Full ms
[50.00-2000.00]
Relative Abundance
m/z

1-(2-cyclopropyl-2-fluorovinyl)-4-nitrobenzene 3ah

^{1}H NMR Spectrum ($CDCl_3$, 300 MHz)

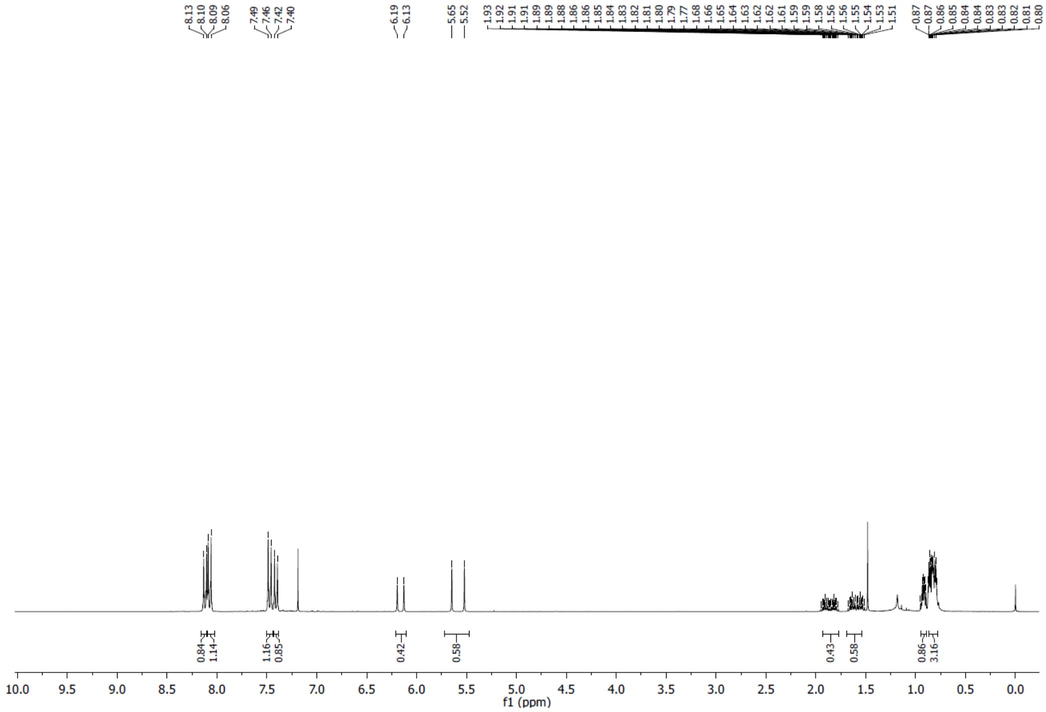

^{13}C ($CDCl_3$, 75 MHz)

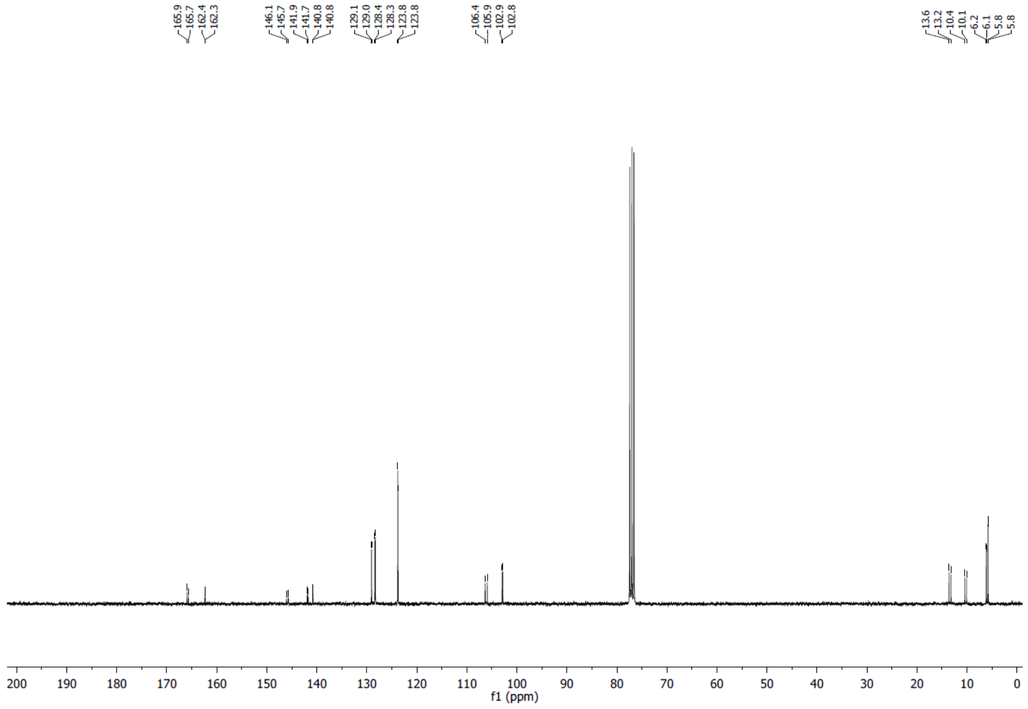

HRMS-spectrum

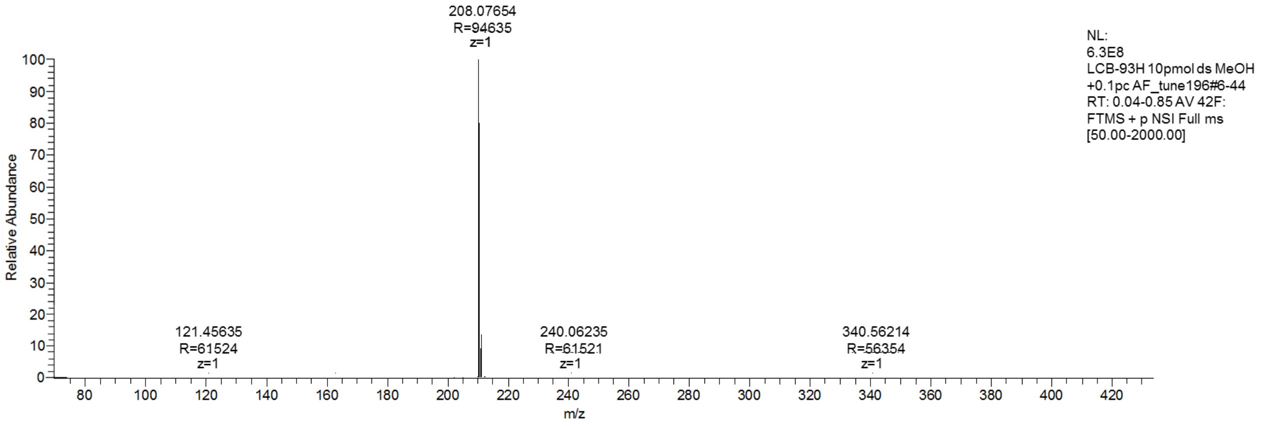

Supplement: Supplementary file 1 [file molecules-25-05532-s001.pdf]
